# Genomic divergence, local adaptation, and complex demographic history may inform management of a popular sportfish species complex

**DOI:** 10.1002/ece3.9370

**Published:** 2022-10-05

**Authors:** Joe C. Gunn, Leah K. Berkman, Jeff Koppelman, Andrew T. Taylor, Shannon K. Brewer, James M. Long, Lori S. Eggert

**Affiliations:** ^1^ Division of Biological Sciences University of Missouri Columbia Missouri USA; ^2^ Missouri Department of Conservation Columbia Missouri USA; ^3^ Department of Biology University of Central Oklahoma Edmond Oklahoma USA; ^4^ U.S. Geological Survey, Alabama Cooperative Fish and Wildlife Research Unit, School of Fisheries, Aquaculture, and Aquatic Sciences Auburn University Auburn Alabama USA; ^5^ U.S. Geological Survey, Oklahoma Cooperative Fish and Wildlife Research Unit, Department of Natural Resource Ecology and Management Oklahoma State University Stillwater Oklahoma USA; ^6^ Department of Biology University of North Georgia Dahlonega Georgia USA

**Keywords:** conservation, demography, divergence, diversity, gene flow, local adaptation

## Abstract

The Neosho Bass (*Micropterus velox*), a former subspecies of the keystone top‐predator and globally popular Smallmouth Bass (*M. dolomieu*), is endemic and narrowly restricted to small, clear streams of the Arkansas River Basin in the Central Interior Highlands (CIH) ecoregion, USA. Previous studies have detected some morphological, genetic, and genomic differentiation between the Neosho and Smallmouth Basses; however, the extent of neutral and adaptive divergence and patterns of intraspecific diversity are poorly understood. Furthermore, lineage diversification has likely been impacted by gene flow in some Neosho populations, which may be due to a combination of natural biogeographic processes and anthropogenic introductions. We assessed: (1) lineage divergence, (2) local directional selection (adaptive divergence), and (3) demographic history among Smallmouth Bass populations in the CIH using population genomic analyses of 50,828 single‐nucleotide polymorphisms (SNPs) obtained through ddRAD‐seq. Neosho and Smallmouth Bass formed monophyletic clades with 100% bootstrap support. We identified two major lineages within each species. We discovered six Neosho Bass populations (two nonadmixed and four admixed) and three nonadmixed Smallmouth Bass populations. We detected 29 SNPs putatively under directional selection in the Neosho range, suggesting populations may be locally adapted. Two populations were admixed via recent asymmetric secondary contact, perhaps after anthropogenic introduction. Two other populations were likely admixed via combinations of ancient and recent processes. These species comprise independently evolving lineages, some having experienced historical and natural admixture. These results may be critical for management of Neosho Bass as a distinct species and may aid in the conservation of other species with complex biogeographic histories.

## Graphical abstract

Using over 50,000 SNPs obtained from ddRAD‐seq, we detected genomic divergence between the globally popular and ecologicaly important Smallmouth Bass (*Micropterus dolomieu*) and Neosho Bass (*M.* *velox*) where their native distributions come together in the Central Interior Highlands ecoregion, USA. We also show strong signatures of genetic differentiation among stream populations due to neutral drift within both subspecies, and we detected adaptive divergence within the Boston Mountains ecoregion of the Neosho native range. Despite differentiation, Neosho Bass stream populations exhibit variable demographic histories, with some populations having been admixed recently, and others historically, with Smallmouth Bass. Our findings provide key insights into levels of genomic diversity and eco‐evolutionary forces driving that diversity, which will be critical for establishing effective conservation and management strategies for these economically important species.

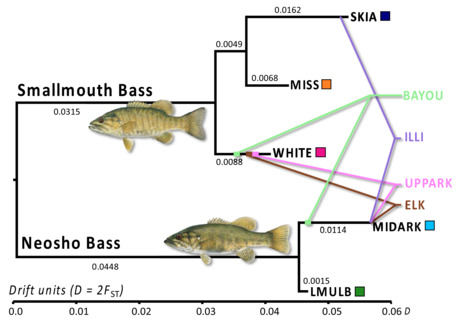

## 1 INTRODUCTION

The convention of classifying organisms into discrete taxonomic units, typically “species,” before they can receive conservation priority (Beheregaray & Caccone, 2007; Isaac et al., 2004) is often politically charged and ignores the biological reality that any arbitrary unit is composed of nested genetic groups. Reciprocally monophyletic lineages at the highest tier of differentiation are made up of metapopulations; metapopulations may consist of many populations; populations are divided into subpopulations and finally into pedigrees. Each of these levels may contain valuable allelic polymorphisms (Lawson, 2013; Præbel et al., 2013) which, in quickly fluctuating environments, could provide the raw material for cladogenesis (Hendry, 2017). Delineating intraspecific variation at the genomic level may therefore aid in biodiversity conservation, especially when that variation is cryptic and perhaps overlooked due to behavioral traits or convergent morphology (Culver et al., 1995; Culver et al., 2009; Schluter, 1996). It is equally crucial to ascertain the eco‐evolutionary context leading to contemporary diversity to predict how species will adapt in the changing world.

Characterizing amounts and patterns of genetic diversity (e.g., allelic richness, allele frequency differentiation, and admixture) and their underlying causal mechanisms (e.g., selection, drift, and gene flow) is challenging for freshwater riverine wildlife. The one‐dimensional, dendritic configuration and abiotic heterogeneity of stream ecosystems, including variable flow rates, depths, temperature gradients, nutrient concentrations, and allochthonous and autochthonous inputs (Barthel et al., 2008; Lytle & Poff, 2004; Vannote et al., 1980), create diverse conditions and restrict movement to relatively narrow corridors within watersheds, setting the stage for population structure (Herdegen et al., 2014; Jacobsen & Hansen, 2005; Puebla, 2009; Ward et al., 1994), local adaptation, and potentially distinct demographic histories among populations. Life history and behavior, such as habitat use, dispersal, and reproduction, may also contribute to eco‐evolutionary dynamics. Fish species that are valued for angling or aquaculture face exceptionally complex environmental pressures because they may also be subjected to human‐mediated introductions (Hohenlohe et al., 2013).

Phylogeography of North American endemic black basses (*Micropterus*) is only partially understood but is likely to have been shaped by both natural and anthropogenic forces. One of the most economically important and globally popular black bass species, the Smallmouth Bass (*M. dolomieu*), occupies a native range extending from the Laurentian Great Lakes in southeastern Canada to the Central Interior Highlands, USA (CIH). Such a broad, ecologically variable distribution and high levels of range‐wide diversity (e.g., Borden & Krebs, 2009) have made it especially difficult to resolve the species' taxonomy. Biologists historically recognized two subspecies, with one encompassing the central and eastern portion of the range (Northern Smallmouth Bass, *M. d. dolomieu*; Hubbs & Bailey, 1940) and another being restricted to the Arkansas River Basin (Neosho Smallmouth Bass, *M. d. velox;* Hubbs & Bailey, 1940). Lack of genome‐wide assessments prevented fine‐scale resolution of differentiation between the subspecies and precluded their designation as independently evolving lineages.

A recent phylogenomic study of the black bass genus provided compelling evidence of genomic divergence between the subspecies (Kim et al., 2022). The authors ultimately elevated the Neosho Smallmouth Bass to species rank (Neosho Bass; *M. velox*), consolidating the Northern Smallmouth Bass as synonymous with Smallmouth Bass (*M. dolomieu*). Recent investigations of morphological and ecological divergence have largely affirmed this taxonomic revision (Brewer et al., 2022; Gunn et al., 2020; Hubbs & Bailey, 1940; Miller & Brewer, 2021, 2022). However, other genetic studies have revealed considerable population structure within both native ranges (Gunn et al., 2020; Long et al., 2021; Stark & Echelle, 1998; Taylor et al., 2018) and substantial, heterogeneous admixture within the Neosho range (Gunn et al., 2020; Taylor et al., 2018), in contrast to the previously assumed scenario of two diverging allopatric lineages. These studies suggest a more complex dichotomy of differentiation and gene flow which varies across clades, streams, and populations, making it challenging to discern evolutionarily significant units (Moritz, 1994) and proceed with conservation and protection priorities.

The most recent time‐calibrated phylogeny of the black basses (Near & Kim, 2021; Kim et al., 2022) dates the split of Smallmouth Bass from its sister clade to between 4 and 6 million years ago. Such an early origin suggests the species was later subjected to Pleistocene glacial fragmentation and climate oscillations, possibly fueling allopatric speciation (Bermingham et al., 1992; Miller, 1965; Near et al., 2003; Puckett et al., 2015; Zink & Slowinski, 1995). Advancement of the glacial front to the last glacial maximum (~22–18 thousand years ago), which coincides with the parapatric convergence of the Smallmouth Bass and Neosho Bass ranges, would have pushed fish into southern refugia (Borden & Krebs, 2009). Later recession may have altered the topography enough to sever river connections, creating opportunities for vicariant speciation. Recession may have also joined rivers through erosion, allowing for dispersal and subsequent gene flow (Berendzen et al., 2008; Near et al., 2001; Near & Keck, 2005; Ray et al., 2006; Zink, 1997). This aligns with the fact that the CIH is an endemism hotspot (Soltis et al., 2006) for freshwater fishes (Cross et al., 1986; Lundberg et al., 2010; McAllister et al., 1986; Robison, 1986) and lends anecdotal support to the possibility of greater inter‐ and intraspecific diversity in black basses in this ecoregion.

In addition to historical geological and ecological shifts, post‐Pleistocene genetic structure may be influenced by contemporary processes. Smallmouth and Neosho Bass populations exhibit inconsistent dispersal and migratory behavior. Some are sedentary (Funk, 1957) due to philopatry and nest‐site fidelity (Ridgway et al., 1991), while others are seasonally potamodromous (Funk, 1957; Gowan et al., 1994; Lyons & Kanehl, 2002); these behaviors may to some degree vary by species (Miller & Brewer, 2021). Smallmouth and Neosho Bass are also extremely economically valuable; hatchery‐raised Smallmouth Bass have been introduced around the globe for recreation, trophy angling, and as a food source (Brewer & Orth, 2015; Iguchi et al., 2004; Robbins & MacCrimmon, 1974; Stark & Echelle, 1998). A genetic strain of Smallmouth Bass derived from the Cumberland River drainage was used to stock the Illinois River system within the Neosho Bass native range in the early 1990s (Taylor et al., 2018). However, this single known stocking event does not adequately explain signatures of substantial, heterogeneous admixture in Neosho streams (Gunn et al., 2020). Unreported or inadvertent introductions may be responsible; otherwise, admixture may be a natural byproduct of stream piracy (Branson, 1963) or recent flooding. Both scenarios substantially impact the genetic integrity of the species in this region.

Elevating the Neosho Bass to species status has profound implications for conservation and management of economically and ecologically valuable populations in a popular sportfish species complex in the CIH. It is therefore critical to understand the extent of divergence, the diversifying mechanisms generating inter‐ and intraspecific diversity, and the homogenizing forces potentially eroding adaptive variation to inform effective strategies for long‐term viability. Genomic sequencing technologies provide the resolution and power to study highly structured populations in complex physical environments which may be susceptible to gene flow. We harnessed reduced representation sequencing (ddRAD‐seq; Peterson et al., 2012) to resolve genomic diversity, local adaptation, and demographic history in the Smallmouth Bass and Neosho Bass, representing some of the world's most popular game fisheries. We specifically examined: (1) phylogenetic hypotheses between and within species; (2) differentiation between and within species at outlier loci; and (3) alternative admixture scenarios using a model‐testing framework, in which we inferred joint demographic histories from population‐specific site frequency spectra. We expected that the Smallmouth and Neosho Bass would be reciprocally monophyletic and that populations would be nested within species. We expected that outlier loci would more strongly differentiate species than populations. Finally, we expected that the genetic architecture of all admixed populations within species would be best explained by very recent gene flow, implicating anthropogenic introductions.

## 2 MATERIALS AND METHODS

### 2.1 Sample collection and genomic DNA preparation

**FIGURE 1:**
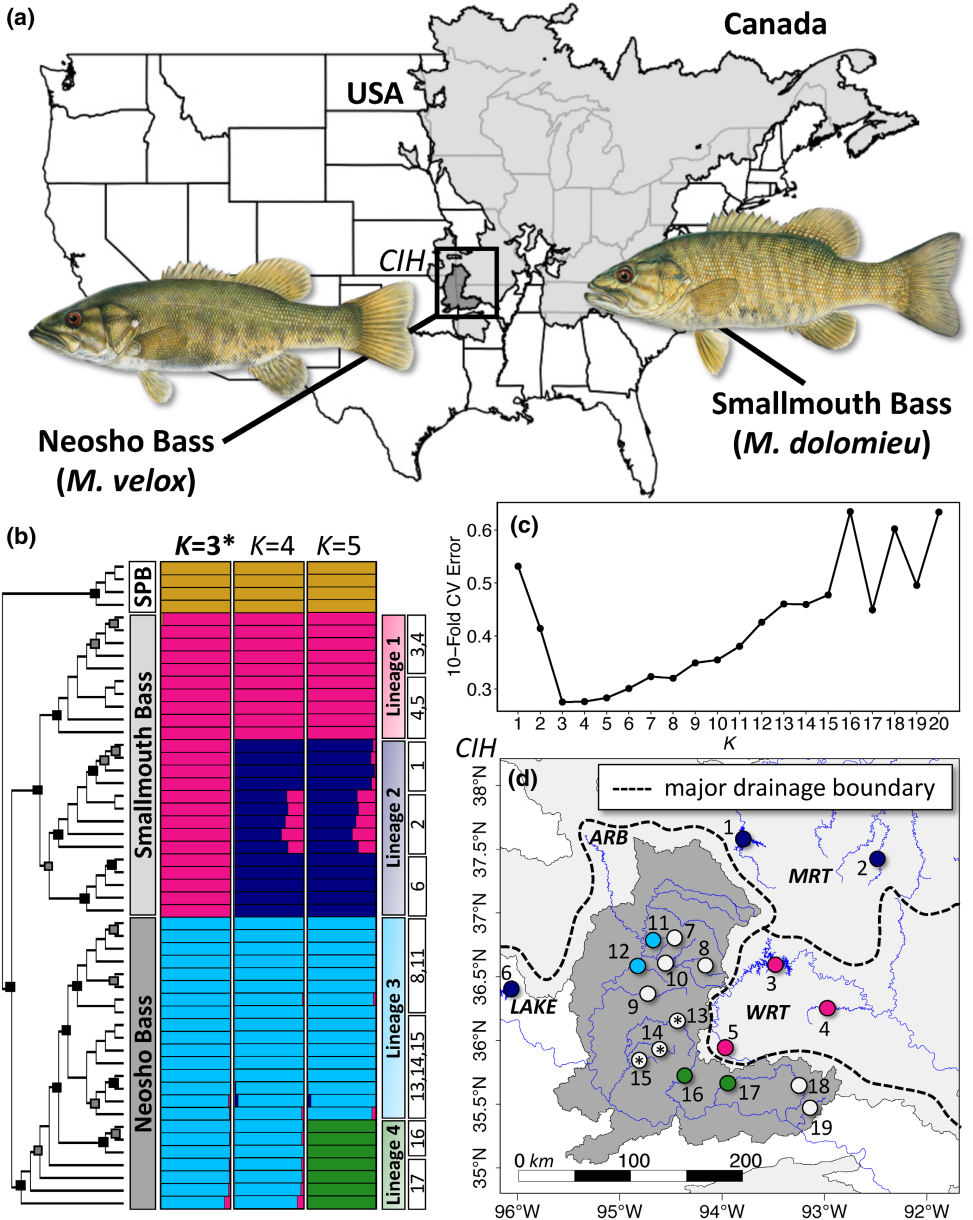
Species geographic ranges, sampling sites, and distinct evolutionary lineages. (a) Native ranges of the Smallmouth Bass (*Micropterus dolomieu*; light gray) and the Neosho Bass (*M. velox*; dark gray), with representative illustrations. (b) Maximum‐likelihood phylogeny for putatively pure (*p*‐Pure) Spotted Bass, Smallmouth Bass, and Neosho Bass, with black and gray boxes at nodes indicating 100% and > 90% bootstrap support, respectively, and population structure results for *K* = 3, *K* = 4, and *K* = 5, with major lineages and sample sites labeled corresponding to individual samples. (c) 10‐fold cross‐validation error results for admixture analysis. (d) Sampling sites (numbered as in Table 1) within the Central Interior Highlands (CIH) for Smallmouth and Neosho Bass colored by distinct evolutionary lineages. Sites of putative admixed origin based on preliminary admixture analysis (*p*‐Admixed) are indicated as white circles; empty white circles indicate sites where all individuals were of putatively admixed origin, and stars indicate sites where nearly all individuals were of putatively admixed origin.

We obtained 95 black bass samples, representing the Neosho Bass (*N* = 66) from 13 streams throughout the Arkansas River Basin (ARB), and the Smallmouth Bass (*N* = 25) from three tributaries of the White River (WRT), two tributaries of the Missouri River (MRT), and Skiatook Lake (LAKE), an impoundment in northeastern Oklahoma situated outside the native range of Smallmouth Bass that was stocked with a hatchery‐reared strain colloquially known as “Tennessee lake‐strain” sourced from the Cumberland River drainage (CIH; Figure 1a and Table 1; Table S1; Gunn et al., 2020). For phylogenomic comparison, we used four Spotted Bass (*M. punctulatus*) from the ARB as an outgroup (Table 1). Including Spotted Bass, we sampled from 20 total stream or impoundment sites.

**TABLE 1:** Sampling sites and associated sample sizes for 25 Smallmouth Bass (*Micropterus dolomieu*), 66 Neosho Bass (*M. velox*), and 4 Spotted Bass (*M. punctulatus*; SPB) from three river drainages (MRT, WRT, and ARB) and one Tennessee Lake‐strain‐stocked lake population (LAKE) before data filtering.

Site ID	Site name	Taxon	Drainage	*N*
1	Stockton Lake	Smallmouth Bass	MRT	4
2	Big Piney River, MO	Smallmouth Bass	MRT	5
3	Tablerock Lake	Smallmouth Bass	WRT	4
4	Crooked Creek	Smallmouth Bass	WRT	4
5	White River	Smallmouth Bass	WRT	3
6	Skiatook Lake	Smallmouth Bass	LAKE	5
			*Total Smallmouth Bass*	25
7	Buffalo Creek	Neosho Bass	ARB	6
8	Sycamore Creek	Neosho Bass	ARB	2
9	Big Sugar Creek	Neosho Bass	ARB	6
10	Elk River	Neosho Bass	ARB	7
11	Honey Creek	Neosho Bass	ARB	6
12	Spavinaw Creek	Neosho Bass	ARB	6
13	Illinois River	Neosho Bass	ARB	7
14	Baron Fork	Neosho Bass	ARB	6
15	Caney Creek	Neosho Bass	ARB	5
16	Lee Creek	Neosho Bass	ARB	4
17	Mulberry River	Neosho Bass	ARB	4
18	Big Piney Creek, AR	Neosho Bass	ARB	2
19	Illinois Bayou River	Neosho Bass	ARB	5
			*Total Neosho Bass*	66
Not Mapped	Honey Creek, Illinois River, Elk River	Spotted Bass (outgroup)	ARB	4
			*Total Spotted Bass*	4

Abbreviations: ARB, Arkansas River Basin; MRT, Missouri River Tributaries; WRT, White River Tributaries.

We extracted high molecular weight DNA (*g*DNA) from ~25 mg fin clips excised from the upper caudal fin using the DNeasy Blood and Tissue kit (QIAGEN, Germantown, MD). Fin clips were coarsely chopped with sterile razor blades, digested with proteinase K, and treated with 4 μl RNase‐A. Extracts were diluted to ~20 ng/μl with ddH_2_O, arranged on a 96‐well plate in 50 μl (~100 μg *g*DNA) aliquots and stored at −20°C before library preparation. We included a single, no‐DNA negative control sample containing only ddH_2_O.

### 2.2 Library preparation and sequencing

Library preparation and sequencing for ddRAD‐seq were completed at Floragenex, Inc. (Eugene, OR) according to a modified Sequence‐based Genotyping protocol outlined in (Truong et al., 2012). Approximately 500 ng genomic DNA was digested with *Pst*I and *Mse*I at 65°C for 1 h, followed by ligation of paired‐end P5 *Pst*I and AFLP *Mse*I adaptors at 37°C for 3 h. Unique, 5‐base pair (bp) barcodes were included in the P5 *Pst*I adaptor for individual sample identification. PCR was performed using the parameters listed in (Truong et al., 2012). Samples (*N* = 95) were pooled and sequenced with 1 × 95 bp chemistry on a single lane of the Illumina HiSeq 4000.

### 2.3 Bioinformatic processing

Sequence filtering, clustering, alignment, and assembly were completed by Floragenex, Inc. (Eugene, OR) according to the *RADseq processing and variant detection pipeline* (Lozier, 2014). Clusters from the sample with the highest number of unique clusters (AR21; Table S1) were used to assemble a de novo reference sequence, which was aligned back to itself to minimize paralogs and to which all Smallmouth and Spotted Bass individuals were aligned (Lozier, 2014).

### 2.4 SNP discovery and filtering

Clusters were processed into RAD tags (95 bp sequences), and single‐nucleotide polymorphisms (SNPs) were called using samtools *v*.0.1.16 (Li et al., 2009) along with custom scripts at Floragenex, Inc. employing the Unified Genotyper within the Genome Analysis Tool Kit (gatk *v*.4.0.1.1; Depristo et al., 2011). The resulting dataset was converted to variant call format (VCF). Subsequent data filtering and subsetting were conducted in vcftools *v*.0.1.16 (Danecek et al., 2011).

Variants were initially filtered based on individual read depth; only sequences with a minimum of 15X coverage were retained. We removed samples from the dataset that had greater than 20% missing genotype calls across SNPs. We then removed SNPs with phred quality scores less than 20 (Liao et al., 2017) and greater than 20% missing genotype calls across samples (e.g., Lavretsky et al., 2019). We then generated two datasets, one in which RAD tags were thinned to retain a single SNP (to reduce the likelihood of linkage between variants in phylogenomic analyses), and one in which RAD tags were not thinned (for fine‐scale population delimitation requiring multiple SNPs per RAD tag to increase computation power).

For both the thinned and nonthinned datasets, we removed remaining SNPs with minor allele count of two or less (equivalent to a minor allele frequency of ~0.011). We created SNP tables using gatk *v*.4.0.1.1, with which we computed genotype frequencies across samples and SNPs using custom scripts in *R v*.4.0.2 (R Core Team, 2018). To reduce bias due to gene duplication, which is known to have occurred deep in the fish phylogeny (McKinney et al., 2017), we eliminated any remaining paralogs by omitting SNPs that were heterozygous in greater than 45% of samples.

### 2.5 Lineage divergence

We screened the full dataset for individuals of putative admixed ancestry, i.e., those of Smallmouth Bass × Spotted Bass or Neosho Bass × Smallmouth Bass hybrid origin, to limit gene flow influence on the assessment of lineage diversification. Our full VCF file was converted to binary pedigree (BED) format in a high‐contig build of plink *v*.1.90 (Chang et al., 2015). We estimated ancestry proportions (*q*) for individual fish in the program admixture *v*.1.3.0 (Alexander et al., 2009), inferring the optimal number of *K* clusters by minimizing 10‐fold cross‐validation error for *K* = 1–20 (number of stream sites plus one additional cluster for the Spotted Bass outgroup). We used stringent criteria to determine pure or admixed origin: individuals were considered putatively pure if *q* ≥ 0.95 for one inferred cluster at the optimal *K*. (Thongda et al., 2020). While minor cluster membership of 0 ≤ *q* ≤ 0.05 for an individual may indicate historical introgression, we retained individuals in this range for phylogenomic comparison to avoid excessively limiting sample sizes. Other studies have resolved lineage divergence using individuals with minor ancestry of 0 ≤ *q* ≤ 0.25 (Kim et al., 2021). Hybrids of Smallmouth Bass or Neosho Bass with Spotted Bass were removed from downstream analyses. The dataset was separated into two subsets: (1) putatively pure individuals (“*p*‐Pure”) and (2) putatively admixed individuals (“*p*‐Admixed”).

We used the *p*‐Pure dataset to investigate phylogenomic relationships among and within Smallmouth and Neosho Bass. We first assessed allele frequency differentiation in the population structure program admixture, choosing *K* by minimizing 10‐fold cross‐validation error for *K* = 1–20. We conducted a parallel phylogenomic analysis using maximum‐likelihood methods in the snphylo pipeline (Edgar, 2004; Felsenstein, 1989; Lee et al., 2014; Schliep, 2011; Zheng et al., 2012). We ran 10,000 bootstrap replicates (−*b*) using Spotted Bass as an outgroup (−*o*) and setting a linkage disequilibrium threshold (−*l*) of 0.1. A consensus tree was constructed in figtree v.1.4.2 (Rambaut, 2010) and aligned with results from admixture.

### 2.6 Population discovery

To delimit populations and assess connectivity among stream sites, we examined haplotype similarity among Smallmouth and Neosho Bass individuals in fineradstructure *v* .0.3.2 (Malinsky et al., 2018 ). We used our full, nonthinned SNP dataset and concatenated SNPs on the same RAD tag to form haplotypes in the radpainter package (Malinsky et al., 2018). We calculated a co‐ancestry matrix with 100,000 burn‐in steps (−*x*), 100,000 Markov chain Monte Carlo (MCMC) iterations (−*y*), and thinning (−*z*) every 1000 iterations for the full sample set, including *p*‐Pure and *p*‐Admixed individuals. We also calculated co‐ancestry matrices separately for the *p*‐Pure and *p*‐Admixed groups to eliminate bias due to multiple ancestry; results from the separate matrices were used for population delimitation. We used a full hill‐climbing tree‐building method to construct trees, running 10,000 iterations (−*x*), providing no value for the initialization parameter (−*T*). Individuals were collapsed into populations if they formed blocks of high co‐ancestry along the diagonal of the co‐ancestry matrix and if they were monophyletic at deeper nodes in the tree.

### 2.7 Adaptive divergence

We explored the role of adaptive divergence influencing variation among and within species by scanning for outlier SNPs putatively showing high (diversifying selection) or low (balancing selection) differentiation among populations (*p*‐Pure and *p*‐Admixed). Outlier analyses are effective in identifying SNPs that deviate from null expectations of allele frequencies under an island model of migration (Lewontin & Krakauer, 1973). However, they are often prone to high rates of false positives, especially when the studied populations are distributed spatially in one‐dimensional, stepping‐stone arrangements, as is the case for riverine fish species (Bierne et al., 2013; Fourcade et al., 2013). We alleviated bias from potential false‐positive results (Excoffier et al., 2009; Jakobsson et al., 2013; Jost, 2008; Nei & Maruyama, 1975; Robertson, 1975) by comparing outliers from different statistical analyses.

We combined bayescan (Foll & Gaggiotti, 2008) and the *R* package *PCAdapt* (Luu et al., 2017) to search for outlier SNPs. The former uses a Bayesian framework to assign posterior probabilities to candidate SNPs based on high or low *F*_ST_ values, whereas the latter employs principal component analysis (PCA) to identify individual SNPs contributing most substantially to variance along principal component axes. While *F*_ST_‐based methods may be compromised by the presence of hierarchical population structure (Flanagan & Jones, 2017) and genotype frequency correlations (Bierne et al., 2013), PCA‐based methods explicitly account for these factors and may be less likely to generate spurious results (Luu et al., 2017).

We conducted genome scans hierarchically at the black bass species level, among species in the Smallmouth Bass species complex, and among populations within species (Chen et al., 2016) to reduce the effect of population structure on outlier detection. We used our full, thinned SNP dataset, dividing individuals into four groups: (1) All black basses, including Spotted Bass, Smallmouth Bass, and Neosho Bass (2) Smallmouth Bass and Neosho Bass, (3) Neosho Bass only, and (4) Smallmouth Bass only.

For each hierarchical analysis, we used default MCMC parameters in bayescan, retaining only SNPs with logged posterior probability greater than 1.5, deemed “very strong” support for selection (Foll & Gaggiotti, 2008). For *PCAdapt*, we tested *K* = 1–20 principal components (PCs). To determine the optimal number of PCs, we assessed Scree plots and selected the number of PCs based on Cattell's Rule (Luu et al., 2017). We generated *p*‐values for all SNPs, applying a Bonferroni correction for multiple tests. Final sets of outlier SNPs were created by merging candidate outliers from bayescan and *PCAdapt* . To assess neutral differentiation (drift), we also created datasets with only shared neutral SNPs (nonoutliers).

We plotted samples according to our a priori population designations (Miller et al., 2020) at outlier and neutral SNPs for all hierarchical analyses using Discriminant Analysis of Principal Components (DAPC; Jombart et al., 2010) in the *R* package *adegenet v*.2.3.1 (Jombart, 2008; Jombart & Ahmed, 2011). We determined the number of PCs to retain in each analysis by selecting the number of PCs maximizing average assignment success over 30 replicates in cross‐validation using the *xval* function (Jombart, 2008; Jombart & Ahmed, 2011), whereby 90% of samples were used as a training set and 10% of samples were used as a test set. The two discriminant functions explaining most of the variation were retained in each analysis. Adaptive divergence was inferred if populations were nonoverlapping based on only outlier SNPs. Genetic drift was inferred by the absence of overlap based solely on neutral SNPs.

### 2.8 Admixture mapping

Admixture signatures could be due to gene flow or incomplete lineage sorting. We tested for evidence of these processes using mixmapper *v*.2.0 (Lipson et al., 2013, 2014). Populations with shared alleles due to incomplete lineage sorting are inferred as nonadmixed, whereas those with a history of admixture postdivergence are inferred as admixed.

We used the full dataset with all inferred populations and created input files in eigensoft *v*.7.2.1 (Patterson et al., 2006; Price et al., 2006). mixmapper uses physical and genetic linkage to calculate genetic drift after admixture. Since our data were not mapped to a reference genome, we did not have linkage information and therefore did not infer precise divergence and mixing times using the drift units generated. To identify significantly admixed populations and fit them to a scaffold tree, we assumed independence (no genetic or physical linkage) of all SNPs (given that we filtered for one SNP per RAD tag during bioinformatic processing) and therefore generated arbitrarily large physical and genetic distance values for each SNP according to the custom formula:

$$d=\frac{100x-y}{100}\;,$$

where *d* = physical/genetic distance, *x* = numerical label of the RAD tag (1, 2, …), and *y* = nucleotide coordinate of SNPs within RAD tags. Moment statistics were calculated using 1000 bootstrap replicates over 50 cM blocks, and the scaffold tree was constructed using 10,000 data subsets. For populations not included in the scaffold tree, we tested the fit of two‐way admixtures between all pairs of nonadmixed parents (sources), running 100 bootstrap replicates. Significantly admixed populations were used in demographic analyses.

### 2.9 Demographic history

We explored potential demographic scenarios driving observed admixture patterns between *p*‐Pure and *p*‐Admixed populations, testing nine two‐population diversification‐based demographic models (Portik et al., 2017) in 𝛿*a*𝛿*i* *v*.3.1.6 (Diffusion Approximation of Demographic Inference; Figure S2a–i; Gutenkunst et al., 2009 *)*; two‐population models allow for divergence with and/or without migration between focal populations. Candidate scenarios differed in the timing of migration, i.e., ancient or due to recent secondary contact, and directionality of migration, i.e., symmetric or asymmetric. Model descriptions are given in Table S2, and parameters estimated are described in Table S3.

Demographic inference in 𝛿*a* 𝛿 *i* assumes SNPs are unlinked and neutral (Gutenkunst et al., 2009). Thus, we used only neutral SNPs ascertained from adaptive divergence analysis, converting SNP data for each population pair into folded 2D joint site frequency spectra (2D‐JSFS). Sample sizes were projected down to account for missing genotypes, and three sequentially finer extrapolation grid sizes were set for each population pair based on the number of alleles per site per population (2*N*). Parameter estimates for each model were determined through a four‐round perturbed optimization procedure as described in Portik et al. (2017), with custom modifications. In the first optimization round, parameter values were initially chosen at random and likelihood values were calculated over a maximum of 3 iterations per 10 replicates. Parameters for the best‐scoring replicate were then used to initiate the next optimization round, in which likelihood was calculated over a maximum of 5 rounds per 20 replicates in round 2, 10 iterations per 30 replicates in round 3, and 15 iterations per 40 replicates in round 4. We checked for convergence of likelihood estimates across rounds for all models. Best‐scoring replicates in round 4 were used to calculate Akaike's Information Criterion (AIC) scores for each model within each admixed‐parent pair, and ΔAIC was then used for model comparison.

It is theoretically feasible to convert 𝛿*a* 𝛿 *i* parameter estimates to measures of migration rates, divergence times, and population sizes. However, since we do not know the true demographic history of these populations, it is possible none of our candidate models fully explain genetic diversity. Additionally, parameter conversion should be conducted with a bootstrapping procedure to quantify uncertainty (Gutenkunst et al., 2009; Portik et al., 2017), and we have relatively low sample sizes for bootstrapping. For these reasons, we did not interpret parameter estimates directly and instead use them for model selection and comparison only.

## 3 RESULTS

### 3.1 Bioinformatic processing

We obtained ~1.76 billion reads across all samples (mean‐per‐sample = 11,209,561.8; *s.d. =* 2,063,521.9). The de novo reference assembly contained ~12.86 million reads passing quality filters, and these were clustered into 240,085 RAD contigs. An average of 59.2% of reads across all samples aligned successfully to the de novo reference. The full genomic dataset contained 357,123 SNPs. A total of 229,694 SNPs were omitted due to low read depth or phred quality scores below a threshold of 20, leaving 127,023 SNPs before filtering for missing data, linkage, minor allele frequency, and excess heterozygosity.

We removed three samples (GRSPB23, ER05, and BFORK32; Table S1) from the dataset that had greater than 20% missing genotype calls (Figure S1). After all filtering, the final dataset (*N* = 92, 24 Smallmouth Bass, 64 Neosho Bass, and 4 Spotted Bass) contained 50,828 SNPs for downstream analyses.

### 3.2 Lineage divergence

Population structure results for all samples were supported at *K* = 4 by 10‐fold cross‐validation (*CV*_error_ = 0.253; Figure S3), revealing that all Smallmouth Bass were putatively of pure origin, but 64% of all Neosho Bass (*N* = 41) were admixed while 36% (*N* = 23) were putatively pure (Figure S4). One Neosho Bass was identified as a likely Spotted Bass hybrid (BFC10; Table S1) and was removed from downstream analyses. One or more *p*‐Admixed individuals were identified in all but three Neosho Bass sample sites (Sites 8, 11, and 17). In six Neosho sampling sites (Sites 7, 9, 10, 12, 18, and 19), all individuals were putatively admixed (Figure S4).

Subsequent population structure analysis on only *p*‐Pure individuals revealed an optimal *K* = 3 (Figure 1b), with all Spotted Bass (SPB), Smallmouth Bass, and Neosho Bass having 0.95 ≤ *q* ≤ 1.00 to distinct genomic clusters. These three major divisions were supported by the maximum‐likelihood phylogeny produced in snphylo, which showed an initial split between the Spotted Bass and a clade comprising Smallmouth Bass and Neosho Bass with 100% bootstrap support, followed by a later split, with 100% bootstrap support, into two monophyletic groups representing the Neosho and Smallmouth Bass species (Figure 1b). The combination of population structure and phylogenomic inference indicated the presence of distinct lineages within Smallmouth and Neosho Bass. At *K* = 4, two monophyletic lineages, Lineage 1 (deep pink) and Lineage 2 (navy blue) emerged within the Smallmouth Bass (Figure 1b). At *K* = 5, an additional lineage was detected in the Neosho Bass, forming Lineage 3 (sky blue) and Lineage 4 (dark green; Figure 1b). The emergent lineage in Neosho Bass did not form a monophyletic group; however, cross‐validation error values for *K* = 4 (*CV*_error_ = 0.276) and *K* = 5 (*CV*_error_ = 0.283) were within 0.008 of the optimal *K* = 3 (*CV*_error_ = 0.275; Figure 1c). We considered this possible evidence of four diverging lineages within Smallmouth and Neosho Bass.

Distinct genomic lineages were clustered geographically, primarily along watershed boundaries (Figure 1d). In Smallmouth Bass, Lineage 1 consisted of three sampling sites restricted to tributaries of the WRT, while Lineage 2 spanned the MRT and the LAKE site. In Neosho Bass, Lineage 3 consisted of four sites throughout the middle of the ARB, and Lineage 4 comprised two sites in southward‐flowing streams in the Boston Mountains of northern Arkansas, USA in the ARB. Sites with either all or mostly admixed individuals were distributed throughout the ARB (Figure 1d).

### 3.3 Population discovery

**FIGURE 2:**
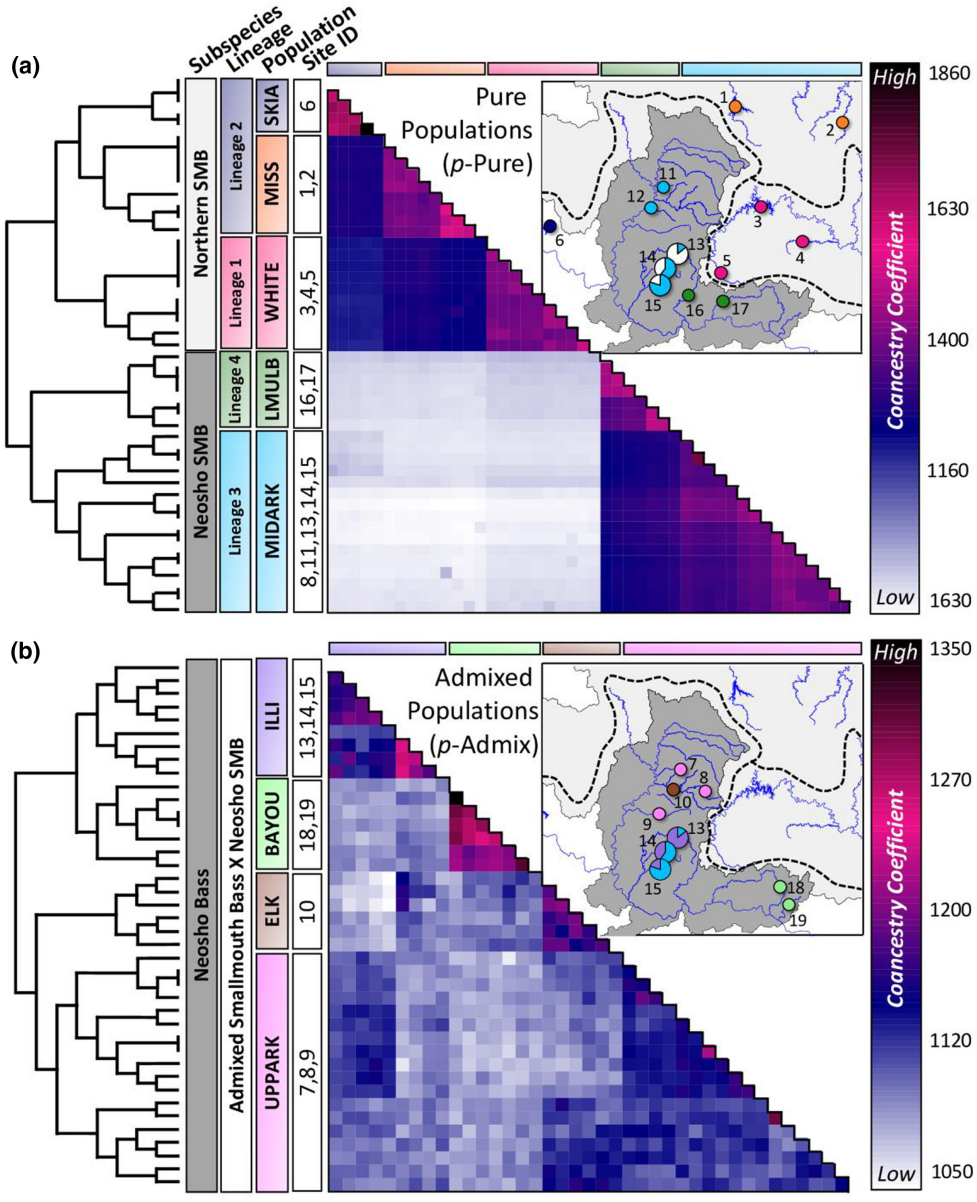
Co‐ancestry and phylogenomic relationships between Smallmouth Bass (*Micropterus dolomieu*) and Neosho Bass (*M. velox*) inferred in fineradstructure for (a) *p*‐Pure samples, and (b) *p*‐Admixed samples. We used the full non‐thinned dataset (98,659 SNPs) to generate SNP haplotypes for co‐ancestry and phylogenomic assessment. Colors within the co‐ancestry matrices reflect the extent of co‐ancestry between adjacent samples, with white and blue representing low co‐ancestry, and pink and black representing high co‐ancestry. Inferred populations are colored on the vertical at left and along the top horizontal for reference, including the hatchery‐stocked LAKE site (SKIA, navy blue), sites within the MRT (MISS, orange), sites within the WRT (WHITE, deep pink), Lee Creek and the Mulberry River (LMULB, dark green), tributaries of the middle ARB (MIDARK, sky blue), the Illinois River system (ILLI, light purple), Illinois Bayou River and Big Piney Creek, AR (BAYOU, light green), the Elk River (ELK, light brown), and tributaries of the upper ARB (UPPARK, light pink). Inset maps show geographic locations of each population; pie charts at Sites 13, 14, and 15 indicate the proportion of individuals assigned as *p*‐Pure (sky blue, a and b) and *p*‐Admixed (white, a; light purple, b). All branches on the phylogenies at left are supported with 100% of bootstrap replicates.

Co‐ancestry analysis of the full sample set did not resolve populations corresponding to rivers, instead showing paraphyly among individuals collected from the same site (Figure S5). Using separate *p‐*Pure and *p*‐Admixed co‐ancestry matrices, we collapsed individuals into nine populations, five of which were found in the *p*‐Pure group (Figure 2a) and four of which were found in the *p*‐Admixed group (Figure 2b). Of the five *p*‐Pure populations, three were detected in the Smallmouth Bass. One coincided exactly with Lineage 1 in the WRT (WHITE), and two were nested in Lineage 2: the LAKE site (SKIA) and the MRT (MISS). The remaining two *p*‐Pure populations belonged to the Neosho Bass and corresponded exactly to Lineage 3 within the middle ARB (MIDARK) and Lineage 4 encompassing Lee Creek and Mulberry River (LMULB; Figure 2a).

Three of four *p*‐Admixed populations formed monophyletic groups adhering to proximate stream sites: the Elk River (ELK), the Illinois River system, including the Illinois River, Caney Creek, and Baron Fork Creek (ILLI), and the Illinois Bayou River and Big Piney Creek, AR (BAYOU; Figure 2b). The fourth *p*‐Admixed population, encompassing streams in the upper ARB (UPPARK), including Big Sugar Creek, Buffalo Creek, and Spavinaw Creek, consisted of two monophyletic clades that did not correspond to stream sites and which did not exhibit high ancestry. Thus, these two clades were grouped as a single population. All nodes in the tree for *p*‐Pure and *p*‐Admixed were confirmed at 100% bootstrap support (Figure 2a,b).

### 3.4 Adaptive divergence

Scanning our SNPs with bayescan across all black bass samples revealed three candidate SNPs under very strong balancing selection (low *F*_ST_) and 50,825 neutral SNPs (Figure S6a). Among Smallmouth and Neosho Bass samples, we found 703 candidate SNPs under diversifying selection and 50,125 neutral SNPs (Figure S6b). Among Neosho Bass only, we found 32 candidate diversifying SNPs and 50,796 neutral SNPs (Figure S6c). Among Smallmouth Bass only, only six candidate SNPs were found to be under substantial selection, and we found 50,822 neutral SNPs (Figure S6d).

In *PCAdapt*, we retained two PCs for analysis with all black bass samples (Figure S7a,b), three PCs for analysis with Smallmouth and Neosho Bass samples (Figure S7c,d), four PCs for analysis with Neosho Bass only (Figure S7e,f), and three PCs for analysis with Smallmouth Bass only (Figure S7g,h) according to the underlying population structure. Outlier SNPs were called based on their loading on retained PCs, with those contributing to most of the variation on a given PC being classified as significant.

In *PCAdapt*, we detected 16,358 candidate outlier SNPs and 34,466 neutral SNPs for all black bass samples. Among Smallmouth and Neosho Bass samples, we detected 1006 candidate diversifying SNPs and 41,871 neutral SNPs. Among Neosho Bass only, we detected 1304 candidate diversifying SNPs and 35,041 neutral SNPs. Among Smallmouth Bass only, we detected 1518 diversifying SNPs and 25,662 neutral SNPs. In each analysis, some SNPs were removed before *p*‐value calculation, because they did not meet the minor allele frequency threshold of 0.01 for that group of samples.

**FIGURE 3:**
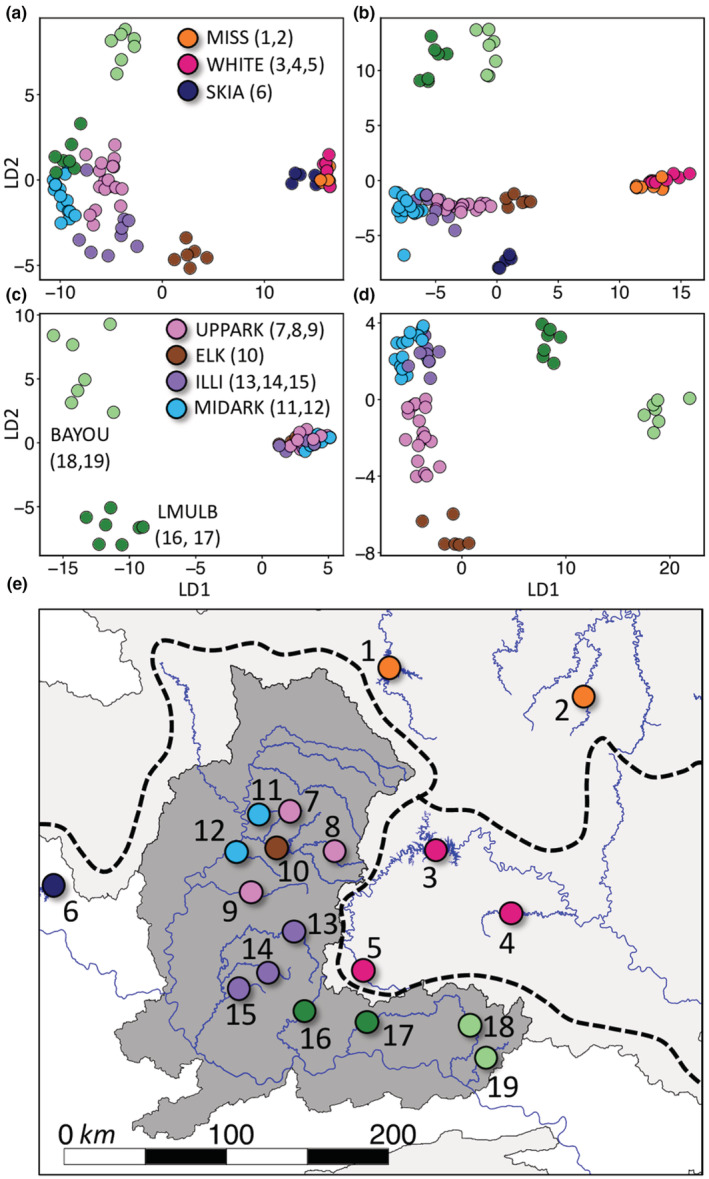
Discriminant Analysis of Principal Components (DAPC) results for (a) outlier (156) and (b) neutral (41,324) SNPs shared by bayescan and pcadapt for all Smallmouth Bass (*Micropterus dolomieu*) and Neosho Bass (*M. velox*) samples; (c) outlier (29) and (d) neutral (35,038) SNPs shared by bayescan and pcadapt for Neosho Bass only. (e) Geographic location of populations.

There were no shared outlier SNPs between bayescan and *PCAdapt* for all black bass samples or Smallmouth Bass only, so we could not reliably consider any SNPs to be contributing to local adaptation. However, among Smallmouth and Neosho Bass samples, we found 156 SNPs with outlier *F*_ST_ values shared between the two models, which drove tight clustering of Smallmouth Bass separately from Neosho Bass as well as clustering of two separate Neosho populations (BAYOU and ELK; Figure 3a; Figures S8a, S9a,b). We also found 41,324 shared neutral SNPs, indicating neutral species differentiation, at the drainage level within the Smallmouth Bass, and between Neosho Bass populations (Figure 3b; Figures S8b, S9c,d). Among Neosho Bass, we detected 29 shared diversifying SNPs which drove strong divergence between the two populations in the Boston Mountains (LMULB, dark green; BAYOU, light green) and between the Boston Mountains and all other Neosho populations (Figure 3c; Figures S8c, S9e,f). We detected 35,038 neutral SNPs, indicative of strong genetic drift among populations (Figure 3d; Figures S8d, S9g,h). Geographic location of populations are given for reference in Figure 3e.

### 3.5 Admixture mapping

**FIGURE 4:**
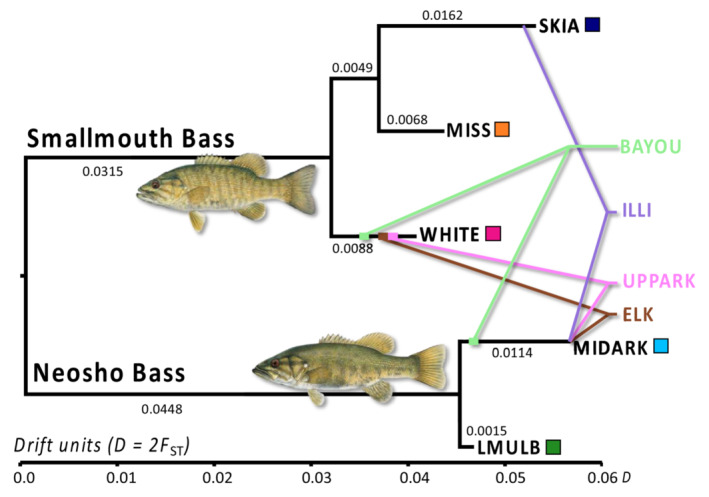
Admixture drift tree for Smallmouth Bass (*Micropterus dolomieu*) and Neosho Bass (*M. velox*) populations inferred in mixmapper. Black labels plotted on the tips of the scaffold tree with corresponding‐colored squares represent pure, unadmixed populations (as determined by a two‐way *f*_3_ test). Colored and shadowed branches and labels mapped onto the scaffold tree represent significantly admixed populations originating from their respective parents. The scale for branch lengths is in drift units (*D*) in which *D* is roughly equal to 2*F*_ST_.

All *p*‐Pure populations (WHITE, MISS, SKIA, MIDARK, and LMULB) were found to be nonadmixed and formed the branch tips of the admixture tree (Figure 4). All *p*‐Admixed populations (ELK, ILLI, UPPARK, and BAYOU) were significantly admixed based on *f*_3_ statistics (Table S4). All admixed populations were inferred to be parented by the MIDARK population of Neosho Bass. The ELK, BAYOU, and UPPARK populations were all admixed with the WHITE population of Smallmouth Bass lineage (Table 2 and Figure 4). The ILLI population was admixed with the SKIA population (Table 2 and Figure 4). In each case, sources were inferred with 100% bootstrap support (Table 2).

**TABLE 2:** Admixed populations of Neosho Bass (*Micropterus velox*), their inferred Smallmouth Bass (*M. dolomieu*) and Neosho Bass parent populations, and parameters inferred from two‐population tests in mixmapper

Adm. Pop	Parent 1	Parent 2	BS	resnorm	alpha	Branch 1 Loc	Branch 2 Loc	Mixed drift
ELK	MIDARK	WHITE	100	1.19E‐06	0.509–0.519	0.011–0.011/0.011	0.005–0.006/0.009	0.001–0.001
ILLI	MIDARK	SKIA	100	1.23E‐06	0.786–0.793	0.011–0.011/0.011	0.015–0.015/0.015	0.000–0.001
BAYOU	MIDARK	WHITE	100	9.12E‐07	0.705–0.720	0.001–0.002/0.011	0.003–0.004/0.009	0.005–0.005
UPPARK	MIDARK	WHITE	100	6.56E‐07	0.785–0.792	0.011–0.011/0.011	0.006–0.007/0.009	0.001–0.001

*Note*: BS gives the number of bootstrap replicates supporting the given pair of parents; resnorm gives the residual error for each test; alpha gives the 95% confidence intervals for the proportion of ancestry from Parent 1; Branch 1 Loc and Branch 2 Loc give the positions on branch 1 and branch 2, respectively, of the admixed population; Mixed Drift gives the drift time since admixture occurred.

Abbreviations: Adm. Pop, Admixed Population; BAYOU, Illinois Bayou River; Branch 1 Loc, Branch 1 location on admixture map; Branch 2 Loc, Branch 2 location on admixture map; BS, Bootstrap support (%); ELK, Elk River; ILLI, Illinois River system; MIDARK, Middle Arkansas River Basin; resnorm, normal residual error; SKIA, Skiatook Lake; UPPARK, Upper Arkansas River Basin; WHITE, White River.

### 3.6 Demographic history

**FIGURE 5:**
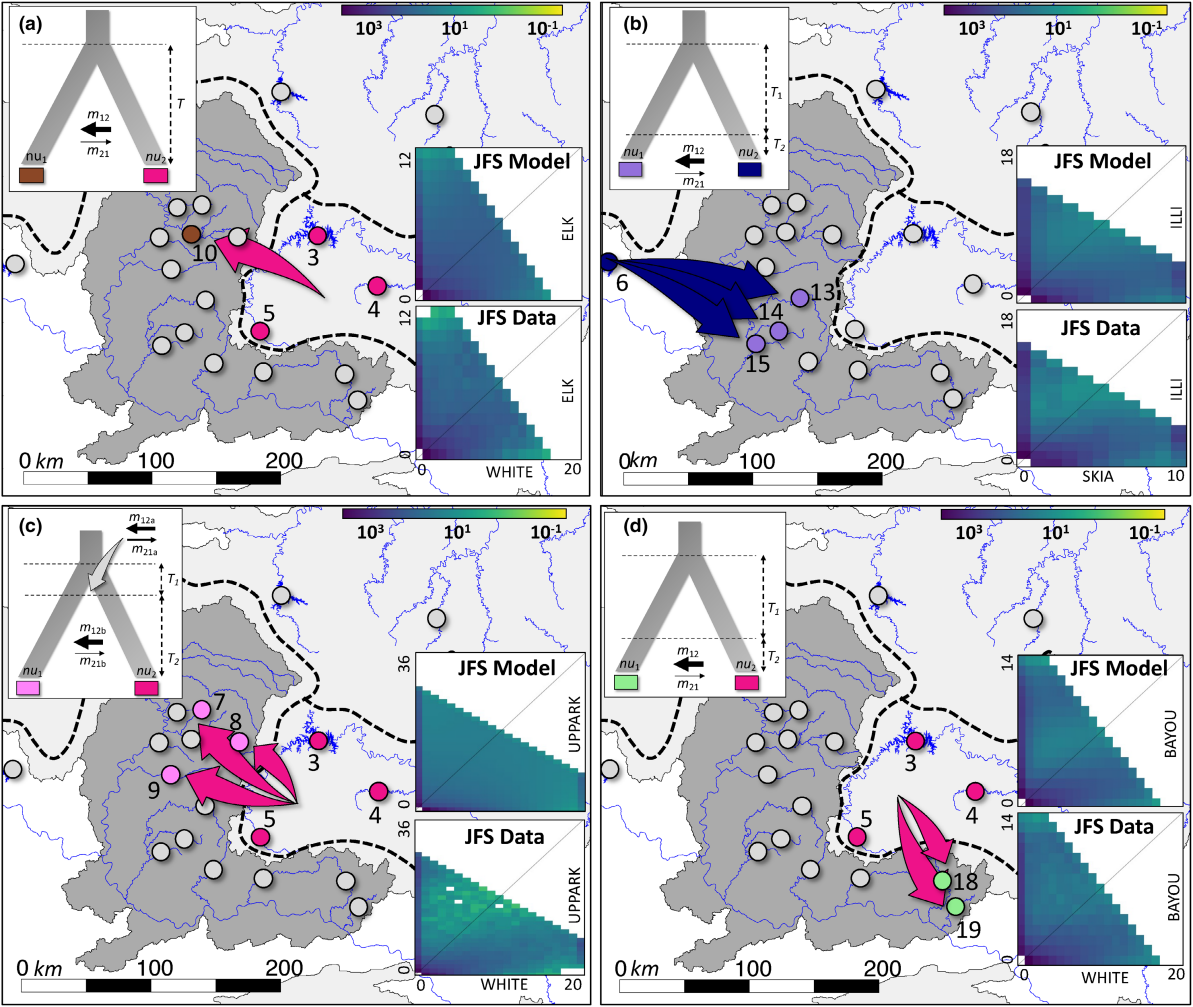
Best‐fitting two‐population demographic models for Smallmouth Bass (*Micropterus dolomieu*) and Neosho Bass (*M. velox*), specifically for (a) ELK and WHITE populations, (b) ILLI and SKIA populations, (c) UPPARK and WHITE populations, and (d) BAYOU and WHITE populations, generated in 𝛿*a* 𝛿 *i*. Model schematics of divergence and migration are shown in the top left‐hand inset; lengths of arrows correspond roughly to the length of time (for *T* parameters) or the rate of migration (for *m* parameters) inferred in 𝛿*a* 𝛿 *i*. The simulated 2D joint site frequency spectrum for the best‐fitting demographic model, and the 2D joint site frequency spectrum for the empirical data, are shown in the top and bottom panels of the lower right‐hand inset, respectively. Residuals representing the closeness of fit of empirical data to each model are given in Figure S14.

All nine demographic models tested in 𝛿*a* 𝛿 *i* were successfully optimized for the ELK and WHITE (Figure S10a–i), ILLI and SKIA (Figure S11a–i), BAYOU and WHITE (Figure S12a‐i), and UPPARK and WHITE (Figure S13a–i) analyses. Parameter estimates and AIC values are provided for all models for each population pair in Table S5. The best‐fitting model (lowest AIC; ∆AIC = 0) for ELK and WHITE was AM (Table S2). Migration rate from WHITE to ELK (*m*_12_ = 4.903) was substantially higher than in the opposite direction (ELK to WHITE; *m*_21_ = 0.026; Table 3 and Figure 5a).

**TABLE 3:** Best‐fitting (∆AIC = 0) two‐population demographic models of admixture history between Smallmouth Bass (*Micropterus dolomieu*) and Neosho Bass (*M. velox*), likelihood, AIC, and parameters output from 𝛿*a* 𝛿 *i*

Pop 1	Pop 2	Model	Parameter estimates										
			*θ*	*nu*_1_	*nu*_2_	*m*_12_	*m*_21_	*m*_12a_	*m*_21a_	*m*_12b_	*m*_21b_	*T*_1_	*T*_2_
ELK	WHITE	AM	6367.420	0.362	0.690	4.903	0.026	‐‐	‐‐	‐‐	‐‐	0.878	‐‐
ILLI	SKIA	SCAM	2854.340	0.962	0.957	3.434	0.276	‐‐	‐‐	‐‐	‐‐	1.143	0.091
UPPARK	WHITE	AM2E	3135.310	0.637	1.508	‐‐	‐‐	1.656	0.096	1.413	0.011	3.076	16.128
BAYOU	WHITE	SCAM	4965.440	0.437	0.867	19.782	0.775	‐‐	‐‐	‐‐	‐‐	0.750	0.019

Abbreviations: *θ*, genetic diversity considering only polymorphic SNPs; AM, divergence with asymmetric migration; AM2E, divergence, asymmetric migration between two distinct epochs; *nu*_1_, size of population 1; *nu*_2_, size of population 2; *m*_12_, continuous migration rate from population 2 to population 1; *m*_12a_, migration rate from population 2 to population 1 during first epoch; *m*_12b_, migration rate from population 2 to population 1 during second epoch; *m*_21_, continuous migration rate from population 1 to population 2; *m*_21a_, migration rate from population 1 to population 2 during first epoch; *m*_21b_, migration rate from population 1 to population 2 during second epoch; SCAM, divergence, isolation, and secondary contact with asymmetric migration; *T*_1_, scaled time between split and migration event; *T*_2_, scaled time between migration event and present.

The best‐fitting model for ILLI and SKIA was SCAM (Table S2). Migration rate from SKIA to ILLI (*m*_12_ = 3.434) was higher than in the opposite direction (ILLI to SKIA; *m*_21_ = 0.276). The estimate for the period of isolation following divergence (*T*_1_ = 1.143) was substantially greater than the time since secondary contact (*T*_2_ = 0.091; Table 3; Figure 5b).

The AM2E model (Table S2) was the most suitable for UPPARK and WHITE, (Table 3; Table S5; Figure 5c). In both the first and second epochs, migration rate from WHITE to UPPARK (*m*_12a_ = 1.656 and *m*_12b_ = 1.413, respectively) was greater than the opposite direction (*m*_21a_ = 0.096 and *m*_21b_ = 0.011). Timing of each epoch with respect to species divergence was variable, although the second epoch (*T*_2_ = 16.128) was estimated to last longer than the first (*T*_1_ = 3.076; Table 3 and Figure 5c).

The most suitable model for BAYOU and WHITE was SCAM (Table S2). Migration rate from WHITE to BAYOU (*m*_12_ = 19.782) was much higher than in the opposite direction (BAYOU to WHITE; *m*_21_ = 0.775). The estimate for the period of isolation following divergence (*T*_1_ = 0.750) was substantially greater than the time since secondary contact (*T*_2_ = 0.019; Table 3 and Figure 5d).

Residual estimates and distributions for all best‐fitting models are given in Figure S14.

## 4 DISCUSSION

Recent studies have attempted a more holistic, multidimensional approach to population genomic investigations which seek not only to reveal the scope of diversity, but also to identify and quantify the strength of evolutionary forces acting on groups of organisms (Bangs et al., 2020; Ebersbach et al., 2020; Portik et al., 2017). By applying this approach and accounting for the many spatially and temporally dynamic processes responsible for phylogeographic patterns, we detected and described the complex genomic diversity of one of the world's most ecologically and economically valuable freshwater sportfish species.

Our phylogenomic and population genomic analyses showed that the Smallmouth and Neosho Bass are highly diverged, forming two monophyletic clades in the CIH. Within the Smallmouth Bass, we found two distinct, monophyletic lineages, one encompassing what was thought to be an intergrade zone between the subspecies in White River drainage, supporting a recent microsatellite‐based study (Gunn et al., 2020). This finding is surprising but makes sense given that endemism in the White River has been observed in other fishes (Roe et al., 2008). A second lineage within the Smallmouth Bass inhabits tributaries of the Missouri River but also includes a reciprocally monophyletic Tennessee lake‐strain population in Skiatook Lake, Oklahoma, USA. Although Skiatook Lake is known to have been stocked with the Tennessee lake‐strain (Taylor et al., 2018), our data confirm it is derived from Smallmouth Bass.

Within the Neosho range, we found a nonadmixed, monophyletic lineage consisting of tributaries throughout the middle of the Arkansas River Basin within the CIH, including Honey Creek, Sycamore Creek, Caney Creek, and Baron Fork, supporting the findings of Taylor et al. (2018) and Gunn et al. (2020). We found an additional distinct lineage in the southward‐flowing streams of northern Arkansas, USA, in the Boston Mountain ecoregion, including the Mulberry River and Lee Creek, which had been noted but poorly resolved by Gunn et al. (2020). The northern Arkansas streams formed a pectinate lineage in the phylogenomic tree despite showing distinct clustering in population structure analysis, perhaps indicating that this may be an early‐diverging lineage. Northern Arkansas may contain segments of an ancestral Neosho Bass population with adaptive allelic variation and could therefore be of high conservation value for the species.

Concordant with their independent evolutionary trajectories, the Smallmouth and Neosho Bass are highly differentiated at multiple outlier SNPs across the genome, which could potentially be explained by directional selection in their local environments. Previously described morphological differences between the species, including head length and number of soft dorsal fin rays (Gunn et al., 2020; Hubbs & Bailey, 1940), have provided support for adaptive diversity. However, this is the first evidence to explicitly show a genomic basis for local selection, and potentially local adaptation, rather than phenotypic plasticity. Additionally, two populations in the Neosho range, one in the Illinois Bayou River and Big Piney Creek and another in Lee Creek and the Mulberry River, were strongly diverged from all other Neosho populations and were differentiated from each other, implicating selection at the lineage and population levels. These results should be interpreted with caution, as there are several other possible drivers of high divergence at individual SNPs, including resistance of gene flow due to chromosomal inversions in regions of low recombination (Kirkpatrick & Barton, 2006), genetic hitchhiking due to selective sweeps (e.g., Kim & Maruki, 2011), purging of deleterious alleles (Pannell & Charlesworth, 2000), demographic history (Lotterhos & Whitlock, 2014), and heterogeneity in recombination rates (Roesti et al., 2012). Fine‐scale genome mapping of candidate selected loci should be conducted to disentangle these effects before local adaptation can be definitively inferred.

Phenotypic differentiation may occur along ecological clines (Conover et al., 2009; Savolainen et al., 2013), and clines can be especially steep among or within rivers (Schlosser, 1991; Vannote et al., 1980). There can be substantial thermal heterogeneity or variation in hydrological factors such as flow rate, depth, and frequency and magnitude of stochastic disturbances, that is, flooding and drought (Barthel et al., 2008; Lytle & Poff, 2004). Thus, fish in different populations may be adapted to specific combinations of variables (Aitken et al., 2008; Davis & Shaw, 2001; Franks & Hoffmann, 2012), and, at the genomic level, such adaptations may be highly polygenic. Both the Illinois Bayou River and Big Piney Creek flow southward through the Boston Mountains of northern Arkansas, USA, before emptying into the Arkansas River. These streams are warm and flow along steep topographical gradients; water temperatures may exceed 30°C (Hafs et al., 2010) in summer, approaching the critical swimming maximum temperature (35°C) for fry (Larimore & Duever, 1968). Although many streams in the Neosho range flow continuously (Robison & Buchanan, 1984), Boston Mountains streams are not spring‐fed and partially dry during summer and autumn months (Hines, 1975), leaving only deep, isolated pools for refuge (Hafs et al., 2010). Neosho Bass in the Boston Mountains may therefore experience occasional isolation and may be well‐adapted to extreme temperature and flow regimes, warranting further investigation and conservation actions given climate projection for warmer temperatures and increased drought intensity for the region (Sharma & Jackson, 2008). A comparison of thermal tolerances of juvenile Neosho (90% HDI: 34.93–36.75°C) versus Ouachita Smallmouth Bass (*M. sp*. cf. *dolomieu velox*; 90% HDI: 36.81–38.6°3C) indicated an approximate 2°C difference in thermal tolerances (Brewer et al., 2022). The thermal tolerance of Smallmouth Bass occupying the Boston Mountains is unknown.

We identified many SNPs that were selectively neutral among Smallmouth and Neosho Bass at both the species and population levels, indicating drift. Some Smallmouth Bass individuals are sedentary (Funk, 1957) as a result of philopatry (Ridgway et al., 1991), which may contribute to reproductive isolation between populations and allow for random fixation of distinct alleles. While some fish may also be migratory, when migration occurs, it is typically seasonal (Funk, 1957; Gowan et al., 1994; Lyons & Kanehl, 2002). Humston et al. (2010) found that first‐year juvenile Smallmouth Bass most often move from tributaries to the mainstem river and not in the opposite direction, but we know little about juvenile dispersal after the first year (Barthel et al., 2008). Given nest‐site fidelity (Ridgway et al., 1991) in most populations, bidirectional movement of Smallmouth Bass cannot be ruled out. Regardless, individuals exhibiting the same movement strategies, either sedentary or migratory, are more likely to interbreed than individuals exhibiting different strategies (Barthel et al., 2008). These same life history traits need to be investigated in Neosho Bass to make a valid ecological comparison. Connectivity among populations may also be limited by impoundments. A tagging study in the Elk River basin showed that tagged Neosho Bass in Sycamore Creek, Elk River, and Buffalo Creek did not cross the reservoir‐river interface created by Grand Lake O′ the Cherokee (Miller & Brewer, 2022). Alternatively, movement is likely limited by dams (Taylor et al., 2019) in the Neosho range, creating bottlenecks and strong drift.

We show that the evolutionary history of Smallmouth and Neosho Bass has been strongly influenced by asymmetric gene flow from the Smallmouth Bass range into the Neosho range. Four of our populations, including seven sampling sites, were significantly admixed. The Elk River and some tributaries of the upper and lower Arkansas River Basin had signatures of allele‐sharing with Smallmouth Bass from the White River drainage. The site frequency distribution of the Elk River population best fits a demographic model of divergence followed by continuous migration from the White River lineage. Gene flow in this part of the range could be facilitated from natural, transient reconnections between the Elk River and the White River system, most likely owing to the karst topography. Several studies (Branson, 1963, 1967) have found evidence of stream capture events in the CIH that may have occurred post‐Pleistocene, a timeframe that could qualify as secondary contact with respect to the timing of species divergence. Intermittent periods of high water (flooding) may have also brought temporary stretches of connectivity. Admixture in the upper Arkansas River tributaries was best modeled by differential rates of asymmetric migration over two distinct epochs. While this demographic scenario has a distinct joint site frequency spectrum (Portik et al., 2017), we presume that the upper Arkansas River tributaries and Elk River populations have undergone similar natural processes given their geographic proximity. It is also likely that both populations have been subjected to anthropogenic introductions of Smallmouth Bass, either inadvertently or deliberately to promote angling (Johnson et al., 2009; Rahel, 2004). Varied timing and differential volumes of introduced fish in these two river systems could explain demography in these rivers. However, our inferences should be considered with caution, as we do not know of direct evidence of Smallmouth Bass stocking.

Admixture in both the Illinois River system and the Illinois Bayou and Big Piney Creek population was best explained by divergence with later secondary contact, more strongly implicating recent, anthropogenic introductions. We expected to find the Illinois River system has been subjected to secondary contact, because we know that it is admixed with the Skiatook Lake genomic cluster due to stocking of Lake Tenkiller with the same hatchery strain in the 1990s (Taylor et al., 2018). We were surprised to obtain the same demographic history for the Illinois Bayou and Big Piney Creek population, because we inferred a greater amount of genetic drift following admixture in this part of the range from our admixture mapping results. Although supported gene flow models were consistent with widespread introductions of Smallmouth Bass from local sources, we have very little direct evidence of Smallmouth Bass stocking. Because we cannot determine the timing of secondary contact using the parameters derived in our demographic analysis, more data are needed before implicating recent introductions as the cause of admixture. Analysis of a full Smallmouth Bass genome and associated linkage map is needed to ascertain precise estimates of admixture timing.

### 4.1 Conservation implications

Global rates of species loss, largely owing to anthropogenic habitat degradation and climate change, continue to climb at a scale warranting designation as Earth's sixth mass extinction (Ceballos et al., 2017; Kuipers et al., 2019). The projected consequences of these trends are dire, as biosphere stability depends on the interconnected ecological roles—e.g., productivity, predation, competition, mutualisms, nutrient cycling—of native, locally adapted lineages (De Meester et al., 2016; Pimm & Raven, 2000; Rovito et al., 2009). Freshwater species, particularly vertebrates, are at disproportionate risk of extinction owing to a number of factors, including pollution, habitat destruction, non‐native species introduction, and overexploitation (Reid et al., 2019). There is a mounting urgency to quantify biodiversity so that we can predict and reduce short‐term ecosystem fragility while maximizing long‐term biodiversity resilience.

Smallmouth Bass are keystone apex predators (Scott & Crossman, 1973) and obligate hosts for the larval stage of several freshwater mussels (Haag et al., 1999; Hoffman, 1999). The Neosho Bass and Smallmouth Bass are phylogenetically divergent, potentially locally adapted lineages in the CIH, each likely playing a vital role in top‐down ecosystem function. We have identified six streams in the Arkansas River Basin—Honey Creek, Sycamore Creek, Caney Creek, Baron Fork, Lee Creek, and the Mulberry River—that appear to be of pure genomic origin and may be descendant populations of ancestral Neosho Bass. These populations may be distinct evolutionary units which may harbor adaptive genomic variation for the species and the greater Ozark Highlands ecosystem. More importantly, some populations within the small, geographically isolated Neosho range are significantly admixed, which could dilute this adaptive diversity and constrain intraspecific diversification.

It is urgent to predict the long‐term fitness outcomes of gene flow in Neosho Bass and other taxa subjected to various forms of secondary contact, especially as species introductions continue to increase (Pearson et al., 2021). Gene flow may cause outbreeding depression through epistatic incompatibilities between derived alleles (Bateson, 1909; Dobzhansky, 1934; Muller, 1942) or undermine coadapted gene complexes that have evolved in isolation (Altukhov & Salmenkova, 1987; Goldberg et al., 2005; Moyle et al., 1986). Mixing may also have the opposite effect, facilitating heterosis by alleviating the genetic load of deleterious genes (Alleaume‐Benharira et al., 2006), establishing stable tension zones (Arnold & Martin, 2010), or reversing stochastic loss of heterozygosity when adaptive alleles flow into small, genetically homogenous populations (Fitzpatrick et al., 2016; Hedrick, 1995; Tallmon et al., 2004; Willi et al., 2007). Even under the latter scenario of adaptive introgression, the ultimate result could be the loss of a distinct lineage through genetic swamping.

Neosho Bass are likely experiencing human‐mediated hybridization and introgression due to introductions. Further research to determine whether introduced alleles are adaptive and becoming more prevalent in Neosho populations, potentially leading to genetic swamping, or, alternatively, if they are maladaptive and reducing relative fitness, would help evaluate the species' evolutionary trajectory. Highly complex patterns of diversification and gene flow have likely gone undetected in other species, both terrestrial and aquatic, that have evolved in variable environments and have been subjected to human‐mediated introductions. This study offers a potential road map for conducting future analyses on nonmodel and potentially threatened species and will aid in the preservation of biodiversity.

## AUTHOR CONTRIBUTIONS

**Joseph C. Gunn:** Conceptualization (lead); data curation (lead); formal analysis (lead); investigation (lead); methodology (lead); resources (equal); visualization (lead); writing – original draft (lead); writing – review and editing (lead). **Leah K. Berkman:** Conceptualization (equal); formal analysis (supporting); funding acquisition (supporting); investigation (supporting); methodology (supporting); project administration (supporting); resources (supporting); supervision (supporting); writing – review and editing (equal). **Jeff Koppelman:** Conceptualization (equal); data curation (supporting); formal analysis (supporting); funding acquisition (supporting); investigation (supporting); methodology (supporting); project administration (supporting); resources (supporting); supervision (supporting); writing – review and editing (equal). **Andrew T. Taylor:** Conceptualization (supporting); data curation (equal); formal analysis (supporting); investigation (supporting); methodology (supporting); project administration (supporting); resources (supporting); writing – review and editing (equal). **Shannon K. Brewer:** Conceptualization (supporting); data curation (supporting); formal analysis (supporting); investigation (supporting); methodology (supporting); project administration (supporting); resources (supporting); writing – review and editing (equal). **James M. Long:** Conceptualization (supporting); data curation (supporting); formal analysis (supporting); investigation (supporting); methodology (supporting); project administration (supporting); resources (supporting); writing – review and editing (equal). **Lori S. Eggert:** Conceptualization (equal); data curation (supporting); formal analysis (supporting); funding acquisition (lead); investigation (equal); methodology (supporting); project administration (lead); resources (supporting); supervision (lead); visualization (supporting); writing – review and editing (equal).

## FUNDING INFORMATION

This project was funded by the Missouri Department of Conservation.

## CONFLICT OF INTEREST

The authors declare that they have no conflict of interest.

### DATA AVAILIBILITY STATEMENT

Raw sequencing data (.*fastq.gz*) and sample metadata (*.xlsx*) are available on Zenodo (https://doi.org/10.5281/zenodo.7032495). All code for genomic analyses is available on GitHub (https://github.com/jcg5g9/SMB_Genomics).

## Supporting information


**Appendix S1:** Supporting information
