# Supplementary material for: Genomic divergence, local adaptation, and complex demographic history may inform management of a popular sportfish species complex
Source: Ecol Evol. 2022 Oct 5;12(10):e9370. doi: 10.1002/ece3.9370 (PMC9534746; doi:10.1002/ece3.9370)
Supplement: Supplementary file 1 — Appendix S1: Supporting information [file ECE3-12-e9370-s001.pdf]

Supplemental Information: Genomic divergence, local adaptation, and complex demographic history may inform management of the Smallmouth Bass species complex

Joe C. Gunn, Leah K. Berkman, Jeff Koppelman, Andrew T. Taylor, Shannon K. Brewer, James M. Long, Lori S. Eggert

Supplemental Tables

Suppl. Table 1. Metadata for Smallmouth Bass (*Micropterus dolomieu*), Neosho Bass (*M. velox*), and Spotted Bass (*M. punctulatus*) collected from the Central Interior Highlands (CIH) by the Missouri Department of Conservation (MDC), University of Missouri (MU), Oklahoma State University (OSU), and Arkansas Tech University (ATU).

ID	Source	Drainage	Taxon	Date Collected	Collector	Northing	Easting
1	**Stockton Lake**	Missouri River Tributaries (MRT)	Smallmouth Bass	October, November, 2017	MDC	434043	4167685
	STOCK03						
	STOCK04						
	STOCK05						
	STOCK06						
2	**Big Piney River, MO**	Missouri River Tributaries (MRT)	Smallmouth Bass	September, 2017	MU	585518	4157157
	BP01						
	BP02						
	BP07						
	BP10						
	BP17						
3	**Tablerock Lake**	White River Drainage (WRD)	Smallmouth Bass	October, November, 2017	MDC	471594	4046542
	TBLR01						
	TBLR02						
	TBLR03						
	TBLR04						
4	**Crooked Creek**	White River Drainage (WRD)	Smallmouth Bass	September, 2015	OSU	507007	4009904
	GRSPB34						
	GRSPB35						
	GRSPB36						
	GRSPB37						
5	**White River**	White River Drainage (WRD)	Smallmouth Bass	September, 2015	OSU	507007	4009904
	GRSPB39						
	GRSPB41						
	GRSPB50						
6	**Skiatook Lake**		Smallmouth Bass	May, 2014	OSU	757815	4024174

	SKIA03	Lake-strain Hatchery (LAKE)					
	SKIA04						
	SKIA05						
	SKIA06						
	SKIA09						
7	**Buffalo Creek**	Arkansas River Basin (ARB)	Neosho Bass	April, May, 2016	OSU	358813	4062302
	BC01						
	BC07						
	BC08						
	BC09						
	BC12						
	BC14						
8	**Sycamore Creek**	Arkansas River Basin (ARB)	Neosho Bass	May, 2016	OSU	349193	4070742
	SC06						
	SC37						
9	**Big Sugar Creek**	Arkansas River Basin (ARB)	Neosho Bass	October, 2015	MU	394297	4052947
	MI419						
	MI420						
	MI421						
	MI422						
	MI423						
	MI425						
10	**Elk River**	Arkansas River Basin (ARB)	Neosho Bass	May, 2016	OSU	358576	4054916
	ER05						
	ER17						
	ER20						
	ER30						
	ER33						
	ER35						
	ER44						
11	**Honey Creek**	Arkansas River Basin (ARB)	Neosho Bass	November, 2016	OSU	347033	4045379
	HC38						
	HC40						
	HC41						
	HC42						
	HC43						
	HC44						
12	**Spavinaw Creek**	Arkansas River Basin (ARB)	Neosho Bass	June, 2015	OSU	346210	4020948
	SPVW02						
	SPVW05						

	SPVW07						
	SPVW11						
	SPVW12						
	SPVW14						
13	**Illinois River**	Arkansas River Basin (ARB)	Neosho Bass	September, 2015	MU,	326757	3967482
	GLVR4				OSU		
	NOIS07						
	NOIS08						
	NOIS12						
	NOIS18						
	SPRMO11						
	SPRMO19						
14	**Baron Fork**	Arkansas River Basin (ARB)	Neosho Bass	July, August, 2015	OSU	328745	3971216
	BFORK02						
	BFORK23						
	BFORK24						
	BFORK30						
	BFORK32						
	BFORK49						
15	**Caney Creek**	Arkansas River Basin	Neosho Bass	July, August, 2015	OSU	339905	3967820
	CANEY15						
	CANEY16						
	GRSPB02						
	GRSPB03						
	GRSPB69						
16	**Lee Creek**	Arkansas River Basin (ARB)	Neosho Bass	*NA*	OSU	361138	3936676
	BFC02						
	BFC06						
	BFC10						
	BFC49						
17	**Mulberry River**	Arkansas River Basin (ARB)	Neosho Bass	September, 2017	MU	437524	3948610
	AR16						
	AR18						
	AR19						
	AR21						
18	**Big Piney Creek, AR**	Arkansas River Basin (ARB)	Neosho Bass	September, 2017	MU	478700	3949057
	AR29						
	AR30						
19	**Illinois Bayou River**	Arkansas River Basin (ARB)	Neosho Bass	September, 2017	ATU	*NA*	*NA*
	AT02						
	AT05						

	AT08						
	AT09						
	AT12						
SPB	**Spotted Bass**	Arkansas River Basin (ARB)	Spotted Bass	September, 2015	OSU	326757	396748 2
	GRSPB51						
	GRSPB52						
	SPRMO49						
	GLVR11						

Suppl. Table 2. References (with respect to Figure S2), ID codes, and descriptions of nine two-population diversification demographic models tested for admixed Neosho Bass (*Micropterus velox*) populations in *δaδi.*

Model Ref	Code	Model Description
**a**	NM	Divergence, no migration
**b**	SM	Divergence, continuous symmetric migration
**c**	AM	Divergence, continuous asymmetric migration
**d**	SCSM	Divergence, isolation, secondary contact with symmetric migration
**e**	SCAM	Divergence, isolation, secondary contact with asymmetric migration
**f**	ASM	Divergence, ancient symmetric migration, isolation
**g**	AAM	Divergence, ancient asymmetric migration, isolation
**h**	SM2E	Divergence, symmetric migration between two distinct epochs
**i**	AM2E	Divergence, asymmetric migration between two distinct epochs

Suppl. Table 3. Description of parameters estimated in $\delta a \delta i$.

Parameter	Parameter Description
nu_1	Size of population 1 at present
nu_2	Size of population 2 at present
m_{12}	Continuous migration rate from population 2 to population 1
m_{21}	Continuous migration rate from population 1 to population 2
m_{12a}	Migration rate from population 2 to population 1 during first epoch
m_{21a}	Migration rate from population 1 to population 2 during first epoch
m_{12b}	Migration rate from population 2 to population 1 during second epoch
m_{21b}	Migration rate from population 1 to population 2 during second epoch
T_1	Scaled time between split and ancient migration
T_2	Scaled time between ancient migration and present

Suppl. Table 4. Results of three-population tests for *p*-Admixed populations of Smallmouth Bass (*Micropterus dolomieu*) and Neosho Bass (*M. velox*; UPPARK, ILLI, ELK, BAYOU). Source combinations (Sources 1 and 2) with the most negative f_3 value are the inferred parents of each admixed population (Admixed Pop) and are highlighted in grey for emphasis.

Admixed Pop	Source 1	Source 2	f_3	Standard Error	*z*-score
UPPARK	MIDARK	MISS	-0.01365	0.00017	-78.92
UPPARK	MIDARK	SKIA	-0.01220	0.00017	-72.51
UPPARK	MIDARK	ELK	-0.00490	0.00008	-59.63
UPPARK	MIDARK	WHITE	-0.01446	0.00017	-83.78
UPPARK	MIDARK	BAYOU	-0.00172	0.00010	-16.87
UPPARK	MISS	LMULB	-0.00463	0.00028	-16.27
UPPARK	SKIA	LMULB	-0.00353	0.00030	-11.97
UPPARK	ELK	LMULB	-0.00190	0.00016	-11.81
UPPARK	LMULB	WHITE	-0.00522	0.00027	-19.62
ILLI	MIDARK	MISS	-0.01452	0.00020	-73.98
ILLI	MIDARK	SKIA	-0.01647	0.00021	-80.31
ILLI	MIDARK	ELK	-0.00311	0.00010	-30.07
ILLI	MIDARK	WHITE	-0.01322	0.00019	-68.41
ILLI	MIDARK	BAYOU	-0.00174	0.00010	-17.83
ILLI	MISS	LMULB	-0.00583	0.00026	-22.08
ILLI	SKIA	UPPARK	-0.00209	0.00018	-11.79
ILLI	SKIA	LMULB	-0.00813	0.00028	-28.54
ILLI	ELK	LMULB	-0.00044	0.00015	-2.97
ILLI	LMULB	WHITE	-0.00431	0.00027	-15.92
ELK	MIDARK	MISS	-0.02100	0.00029	-72.97
ELK	MIDARK	SKIA	-0.01820	0.00030	-60.57
ELK	MIDARK	WHITE	-0.02240	0.00024	-94.07
ELK	MISS	UPPARK	-0.01225	0.00021	-57.99
ELK	MISS	ILLI	-0.00959	0.00022	-43.31
ELK	MISS	LMULB	-0.01498	0.00028	-52.68
ELK	MISS	BAYOU	-0.00427	0.00021	-20.61
ELK	SKIA	UPPARK	-0.0109	0.00023	-46.99
ELK	SKIA	ILLI	-0.00485	0.00023	-20.97
ELK	SKIA	LMULB	-0.01254	0.00031	-41.00
ELK	SKIA	BAYOU	-0.00281	0.00025	-11.25
ELK	UPPARK	WHITE	-0.01284	0.00017	-76.54
ELK	ILLI	WHITE	-0.01230	0.00021	-59.69
ELK	LMULB	WHITE	-0.01616	0.00023	-71.61
ELK	WHITE	BAYOU	-0.00511	0.00021	-24.73
BAYOU	MIDARK	MISS	-0.01140	0.00027	-41.99
BAYOU	MIDARK	SKIA	-0.01005	0.00028	-35.50
BAYOU	MIDARK	WHITE	-0.01195	0.00026	-46.28
BAYOU	MISS	LMULB	-0.01053	0.00030	-35.54
BAYOU	SKIA	LMULB	-0.00955	0.00029	-32.58
BAYOU	ILLI	WHITE	-0.00047	0.00024	-1.99
BAYOU	LMULB	WHITE	-0.01087	0.00027	-40.57

Suppl. Table 5. Log-likelihood, Akaike's Information Criterion (AIC), ΔAIC, and parameter estimates for models assessed in *δaδi*.

Model	Log(LIK)	AIC	ΔAIC	X^2	θ	nu_1	nu_2	m_{12}	m_{21}	m_{12a}	m_{21a}	m_{12b}	m_{21b}	T_1	T_2
ELK-WHITE															
**AM**	**-1680.740**	**3371.480**	**0.000**	**2845.700**	**6367.420**	**0.362**	**0.690**	**4.903**	**0.026**	--	--	--	--	**0.878**	--
SCAM	-1686.190	3384.380	12.900	2805.280	10355.270	0.200	0.417	7.872	0.291	--	--	--	--	2.532	0.772
AM2E	-2015.220	4046.440	674.960	3520.880	3682.220	0.870	0.972	--	--	3.766	1.587	1.998	0.250	2.084	0.756
AAM	-2587.250	5186.500	1815.020	5447.180	487.070	6.119	8.419	0.370	0.085	--	--	--	--	25.239	0.416
ASM	-2939.020	5888.040	2516.560	6020.250	507.130	7.755	5.287	0.176	0.176	--	--	--	--	24.728	0.135
SM	-2970.100	5948.200	2576.720	6226.700	2105.330	1.871	1.286	0.643	0.643	--	--	--	--	4.853	--
SM2E	-2983.360	5978.720	2607.240	6331.740	4844.120	0.759	0.576	--	--	5.847	5.847	1.349	1.349	1.023	0.302
SCSM	-3075.980	6161.960	2790.480	6047.350	23253.430	0.150	0.099	8.110	8.110	--	--	--	--	0.166	4.984
NM	-3296.110	6598.220	3226.740	7179.420	6360.550	0.445	0.410	--	--	--	--	--	--	0.087	--
ILLI-SKIA															
**SCAM**	**-1782.330**	**3576.660**	**0.000**	**3050.590**	**2854.340**	**0.962**	**0.957**	**3.434**	**0.276**	--	--	--	--	**1.143**	**0.091**
AM	-3374.100	6758.200	3181.540	7183.260	5031.200	0.405	0.501	1.988	0.035	--	--	--	--	0.998	--
AM2E	-3426.530	6869.060	3292.400	7194.670	8356.110	0.248	0.349	--	--	12.606	0.223	3.296	0.049	1.172	2.678
SCSM	-3995.760	8001.520	4424.860	8600.030	1325.630	2.540	1.121	0.497	0.497	--	--	--	--	4.277	0.578
AAM	-4061.900	8135.800	4559.140	9271.600	5738.750	0.394	0.335	2.780	0.418	--	--	--	--	0.590	0.013
SM	-4362.920	8733.840	5157.180	9799.840	15555.610	0.175	0.086	3.592	3.592	--	--	--	--	2.738	--
SM2E	-4362.890	8737.780	5161.120	9781.380	3482.880	0.780	0.393	--	--	9.060	9.060	0.800	0.800	0.485	5.548
ASM	-4536.060	9082.120	5505.460	10882.700	457.150	6.675	3.124	0.112	0.112	--	--	--	--	27.211	0.118
NM	-5777.170	11560.340	7983.680	14792.680	5681.110	0.312	0.131	--	--	--	--	--	--	0.077	--
UPPARK-WHITE															
**AM2E**	**-6121.950**	**12259.900**	**0.000**	**11792.280**	**3135.310**	**0.637**	**1.508**	--	--	**1.656**	**0.096**	**1.413**	**0.011**	**3.076**	**16.128**
AM	-6144.990	12299.980	40.080	11783.010	1554.960	1.259	3.043	0.687	0.010	--	--	--	--	16.249	--
SCAM	-6215.650	12443.300	183.400	12124.500	10236.050	0.218	0.411	4.126	0.245	--	--	--	--	4.664	3.213
AAM	-6645.750	13303.500	1043.600	13734.960	2276.210	1.062	1.837	0.950	0.044	--	--	--	--	10.348	0.010
SCSM	-7521.570	15053.140	2793.240	17579.200	5409.540	0.600	0.518	2.140	2.140	--	--	--	--	0.588	0.090
SM2E	-7528.020	15068.040	2808.140	18231.120	1798.340	1.913	1.683	--	--	0.062	0.062	0.607	0.607	4.240	0.324
SM	-8651.010	17310.020	5050.120	20386.740	1535.130	2.096	1.702	0.296	0.296	--	--	--	--	9.468	--
ASM	-9020.850	18051.700	5791.800	21859.010	3329.230	0.876	0.782	0.775	0.775	--	--	--	--	8.007	0.011
NM	-12410.300	24826.600	12566.700	37087.760	7016.920	0.332	0.289	--	--	--	--	--	--	0.091	--
BAYOU-WHITE															
**SCAM**	**-1049.790**	**2111.580**	**0.000**	**1127.730**	**4965.440**	**0.437**	**0.867**	**19.782**	**0.775**	--	--	--	--	**0.750**	**0.019**
AM2E	-1853.650	3723.300	1611.720	3005.520	10102.310	0.185	0.411	--	--	0.029	0.571	5.001	0.131	0.322	0.835
AM	-1858.620	3727.240	1615.660	2973.110	15135.790	0.115	0.275	8.156	0.143	--	--	--	--	0.784	--
AAM	-3482.480	6976.960	4865.380	7610.390	6387.160	0.432	0.541	3.647	1.045	--	--	--	--	2.113	0.056
SM	-3498.620	7005.240	4893.660	6995.780	4861.830	0.696	0.594	0.864	0.864	--	--	--	--	1.220	--
ASM	-3506.790	7023.580	4912.000	7058.010	2528.710	1.337	1.147	0.469	0.469	--	--	--	--	4.118	0.010
SM2E	-3541.000	7094.000	4982.420	6893.060	12839.690	0.242	0.210	--	--	8.279	8.279	2.361	2.361	7.109	0.218
SCSM	-3644.410	7298.820	5187.240	6932.470	6202.980	0.480	0.403	1.312	1.312	--	--	--	--	0.060	4.630
NM	-4374.010	8754.020	6642.440	9964.400	6765.700	0.341	0.375	--	--	--	--	--	--	0.112	--

Supplemental Figures

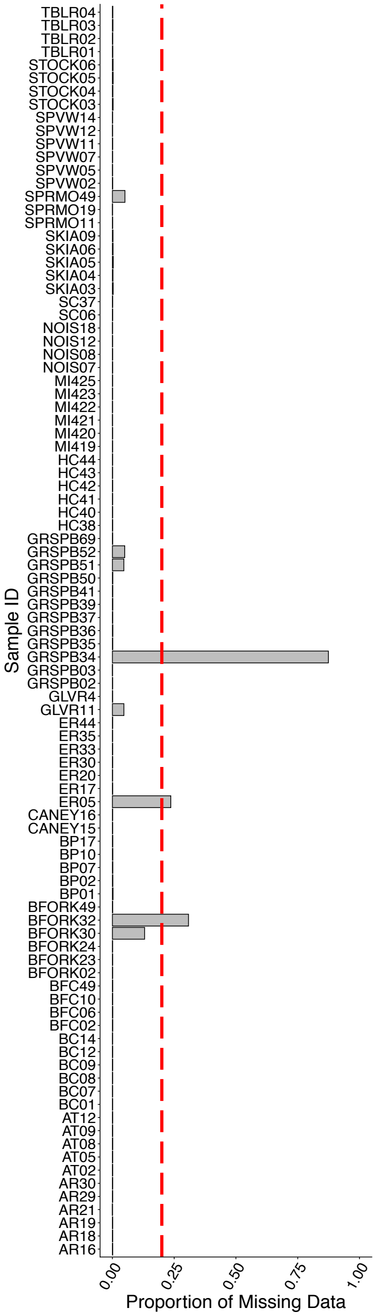

Suppl. Fig. 1. Proportion of missing genotype data per Smallmouth Bass (*Micropterus dolomieu*), Neosho Bass (*M. velox*), and Spotted Bass (*M. punctulatus*) specimen across all single nucleotide polymorphisms (SNPs; $N_{\mathrm{SNPS}} = 127{,}428$) before filtering. The red dotted line indicates the threshold (≤20% missing data) for keeping a sample.

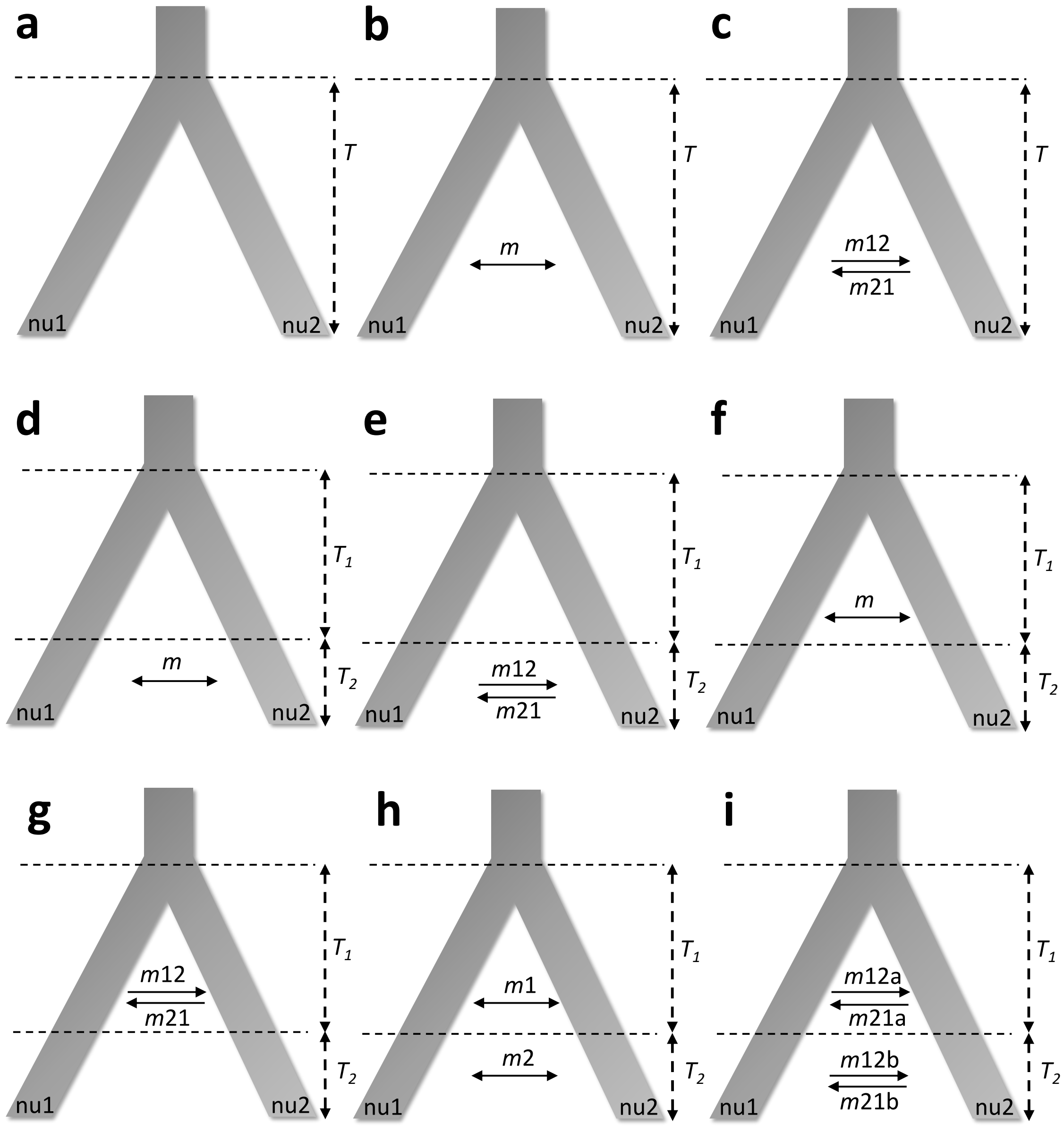

Suppl. Fig. 2. Two-population diversification demographic models for Smallmouth Bass (*Micropterus dolomieu*) and Neosho Bass (*M. velox*) tested in δaδi for each admixed population pair, along with associated parameters. Models were fitted using 2D folded joint frequency spectra and represent (a) population divergence with no migration; (b) divergence with continuous symmetric migration; (c) divergence with continuous asymmetric migration; (d) divergence, isolation, and secondary contact with symmetric migration; (e) divergence, isolation, and secondary contact with asymmetric migration; (f) divergence, ancient symmetric migration, isolation; (g) divergence, ancient asymmetric migration, isolation; (h) divergence, symmetric migration over two distinct epochs; and (i) divergence, asymmetric migration over two distinct epochs.

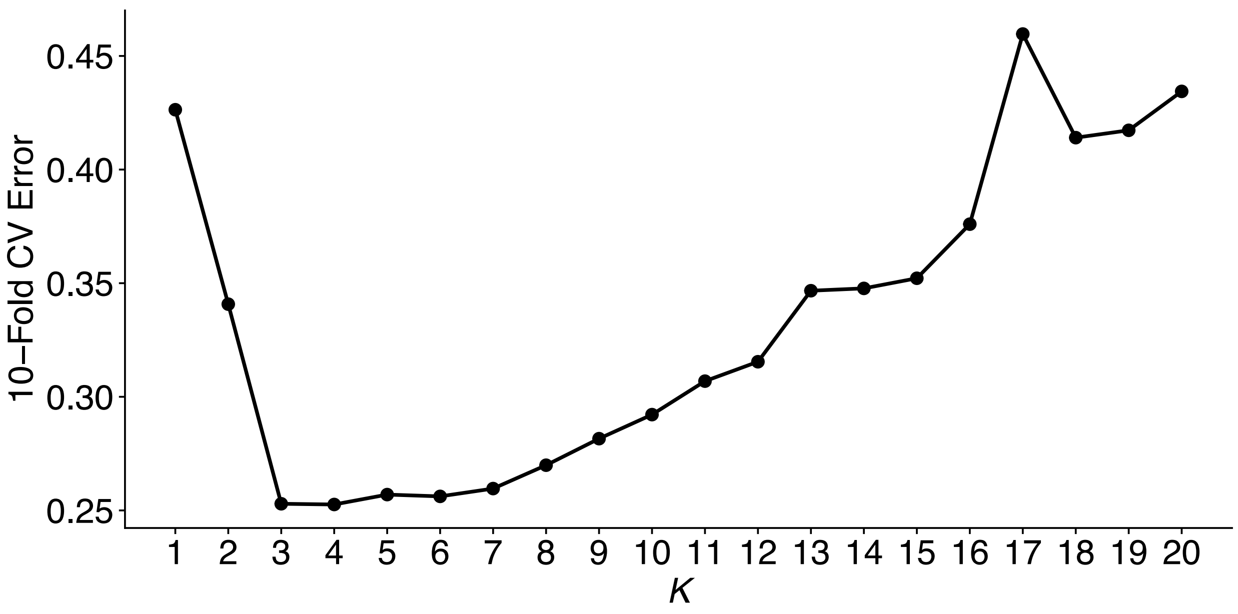

Suppl. Fig. 3. 10-fold cross-validation error results for admixture screening analysis of all Smallmouth Bass (*Micropterus dolomieu*), Neosho Bass (*M. velox*), and Spotted Bass (*M. punctulatus*) samples.

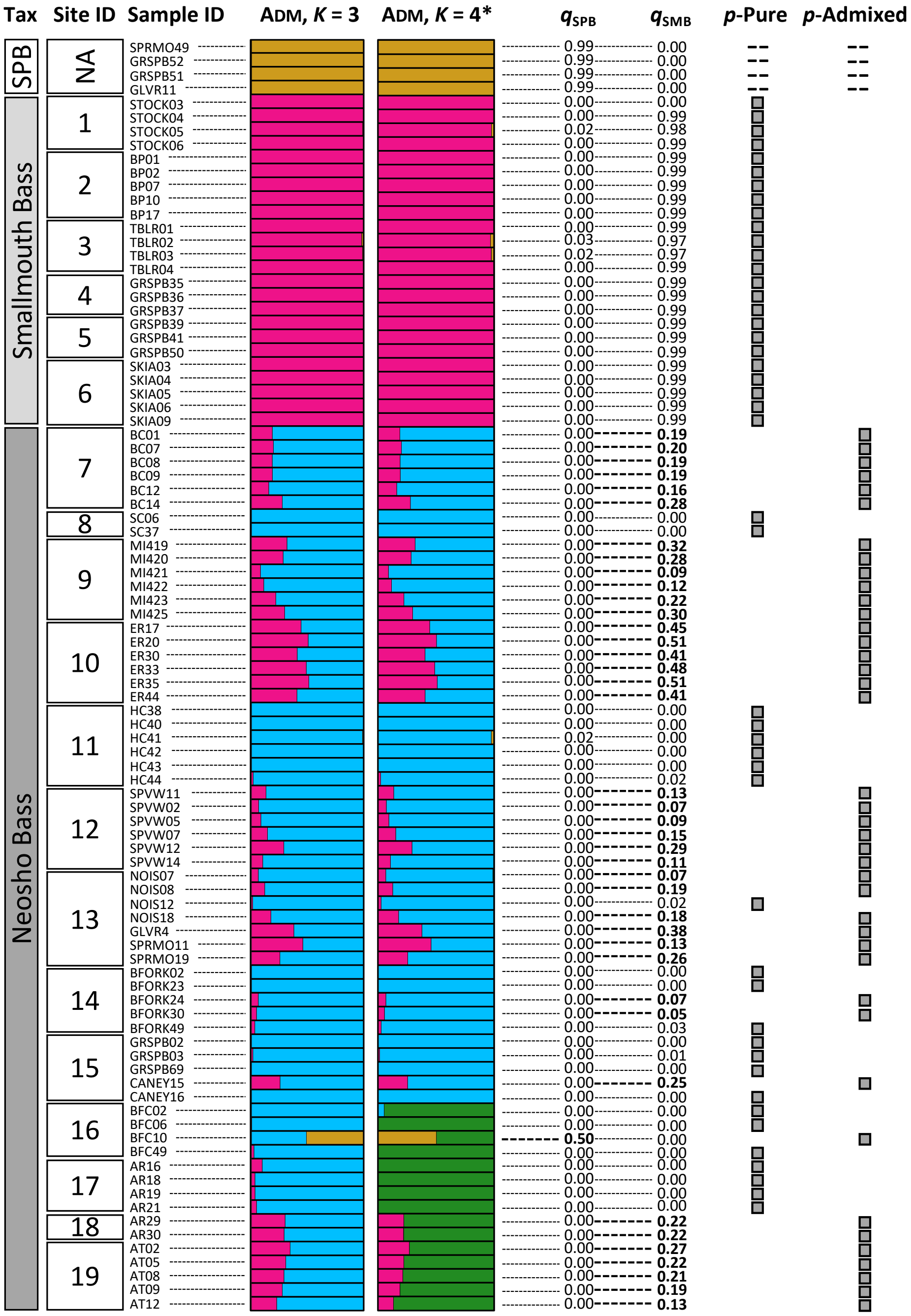

Suppl. Fig. 4. Population structure results for K=4 generated in ADMIXTURE for all retained (N = 92) Smallmouth Bass (*Micropterus dolomieu*; N = 24), Neosho Bass (*M. velox*; N = 63), and Spotted Bass (SPB; *M. punctulatus*; N=4) and all single nucleotide polymorphisms (SNPs) passing quality filters (50,828). Individuals are labeled from left to right by taxon (Tax), Site ID, and Sample ID. Ancestry coefficients for Spotted Bass (q_{SPB}) and Smallmouth Bass (q_{SMB}) are given and connected by thin dotted lines for ease of visualization. A check was placed in the *p*-Pure column if q_{SMB}<0.05 and q_{SPB}<0.05. Otherwise, a check was placed in the *p*-Admixed column, indicated a putatively admixed individual. "NA" indicates that no populations were defined. The K value marked with "*" indicates optimal K based on 10-fold cross-validation.

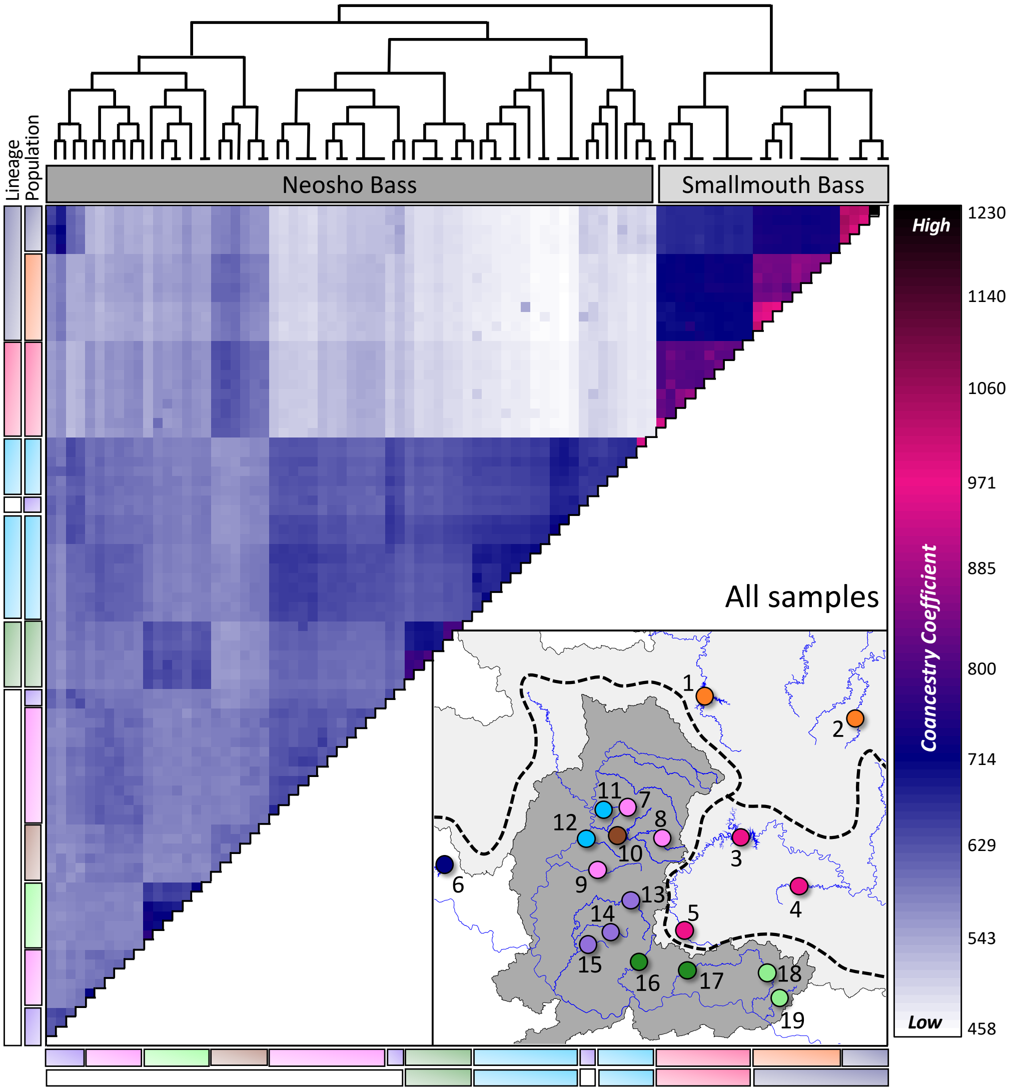

Suppl. Fig. 5. Co-ancestry and phylogenetic relationships for all Smallmouth Bass (*Micropterus dolomieu*) and Neosho Bass (*M. velox*) samples inferred in FINERADSTRUCTURE. using 98,659 single nucleotide polymorphisms (SNPs) and excluding the hybrid BFC10. We used the non-thinned dataset (98,659 SNPs) to generate SNP haplotypes for co-ancestry and phylogenetic assessment. Colors within the co-ancestry matrices reflect the extent of co-ancestry between adjacent samples, with white and blue representing low co-ancestry, and pink and black representing high co-ancestry. Lineages inferred from ADMIXTURE and SNPHYLO are indicated on the left-hand vertical column, and subspecies is designated on the top horizontal. A maximum likelihood tree of relationships is given on the upper horizontal. Inset map gives geographic locations of all populations. All branches on the phylogenies at left are supported with 100% of bootstrap replicates.

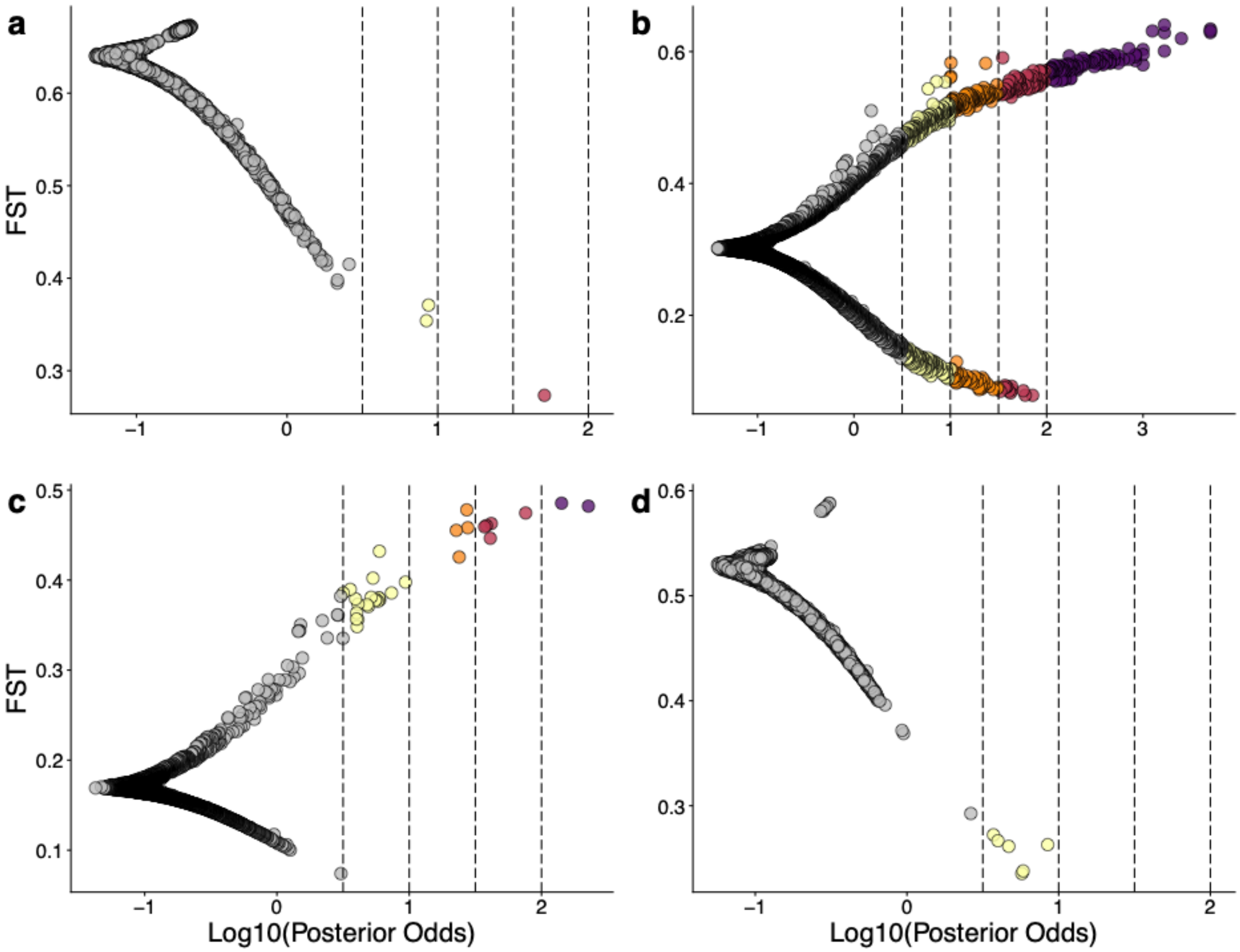

Suppl. Fig. 6. Single nucleotide polymorphisms (SNPs) with outlier F_{ST} detected in BAYESCAN. (a) Outlier SNPs detected across Spotted Bass (*Micropterus punctulatus*), Smallmouth Bass (*M. dolomieu*), and Neosho Bass (*M. velox*) samples; (b) outlier SNPs detected across Smallmouth and Neosho Bass only; (c) outlier SNPs detected across Neosho Bass only; and (d) outlier SNPs detected across Smallmouth Bass only. Log10 of the posterior odds for each SNP are plotted on the x-axis, and associated F_{ST} values are plotted on the y-axis. Dotted lines are plotted on the x-axis at 0.5, 1.0, 1.5, and 2.0, designating posterior odds corresponding to thresholds for substantial support, strong support, very strong support, and decisive support for outlier status, respectively, and dots are colored according to the threshold they meet. High outlier F_{ST} SNPs are under strong diversifying (positive) selection, whereas low outlier F_{ST} SNPs are under strong balancing (stabilizing) selection. SNPs with posterior odds above 1.5, colored in dark red and dark purple, were considered significant for downstream analyses.

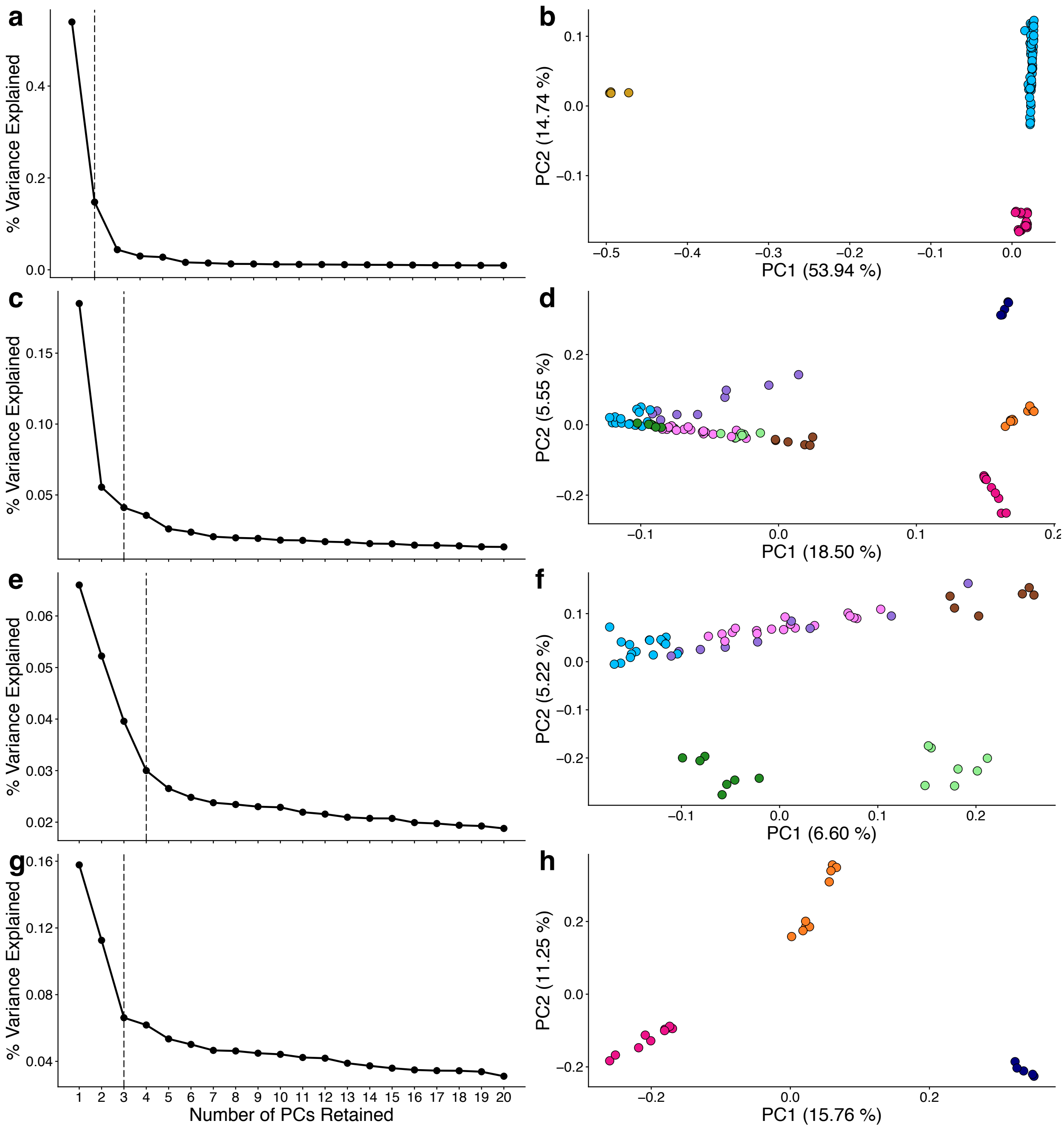

Suppl. Fig 7. Scree plots (left column) and principal components analysis (PCA) score plots (right column) from outlier F_{ST} analysis in *PCAdapt* for all Spotted Bass (*Micropterus punctulatus*), Smallmouth Bass (*M. dolomieu*), and Neosho Bass (*M. velox*) samples combined (a and b), Smallmouth and Neosho Bass samples only (c and d), Neosho Bass samples only (e and f) and Smallmouth Bass samples only (g and h). Dotted lines on the Scree plot indicate the number of PCs used to calculate the PCA shown at right. Only the first 2 PCs (with proportion of explained variance listed for each) are shown. PCA plots were colored by the most prominent level population structure.

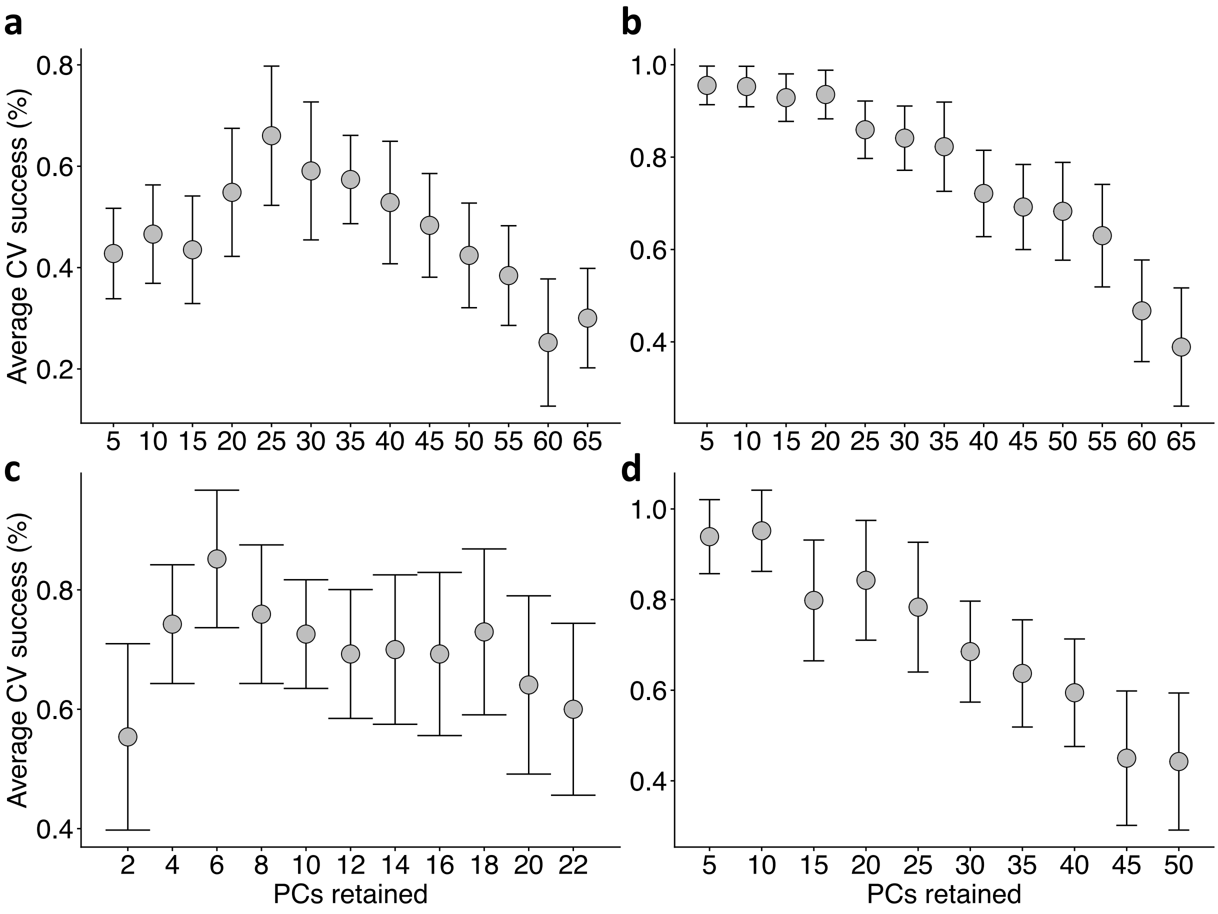

Suppl. Fig. 8. Cross-validation results showing the average proportion of successful individual assignments of Smallmouth Bass (*Micropterus dolomieu*) and Neosho Bass (*M. velox*) to *a priori* populations across varying numbers of retained principal components in discriminant analysis of principal components (DAPC) for (a) outlier single nucleotide polymorphisms (SNPs) in all Smallmouth and Neosho Bass samples; (b) neutral SNPs in all Smallmouth and Neosho Bass samples; (c) outlier SNPs in Neosho Bass samples only; and (d) neutral SNPs in Neosho Bass samples only.

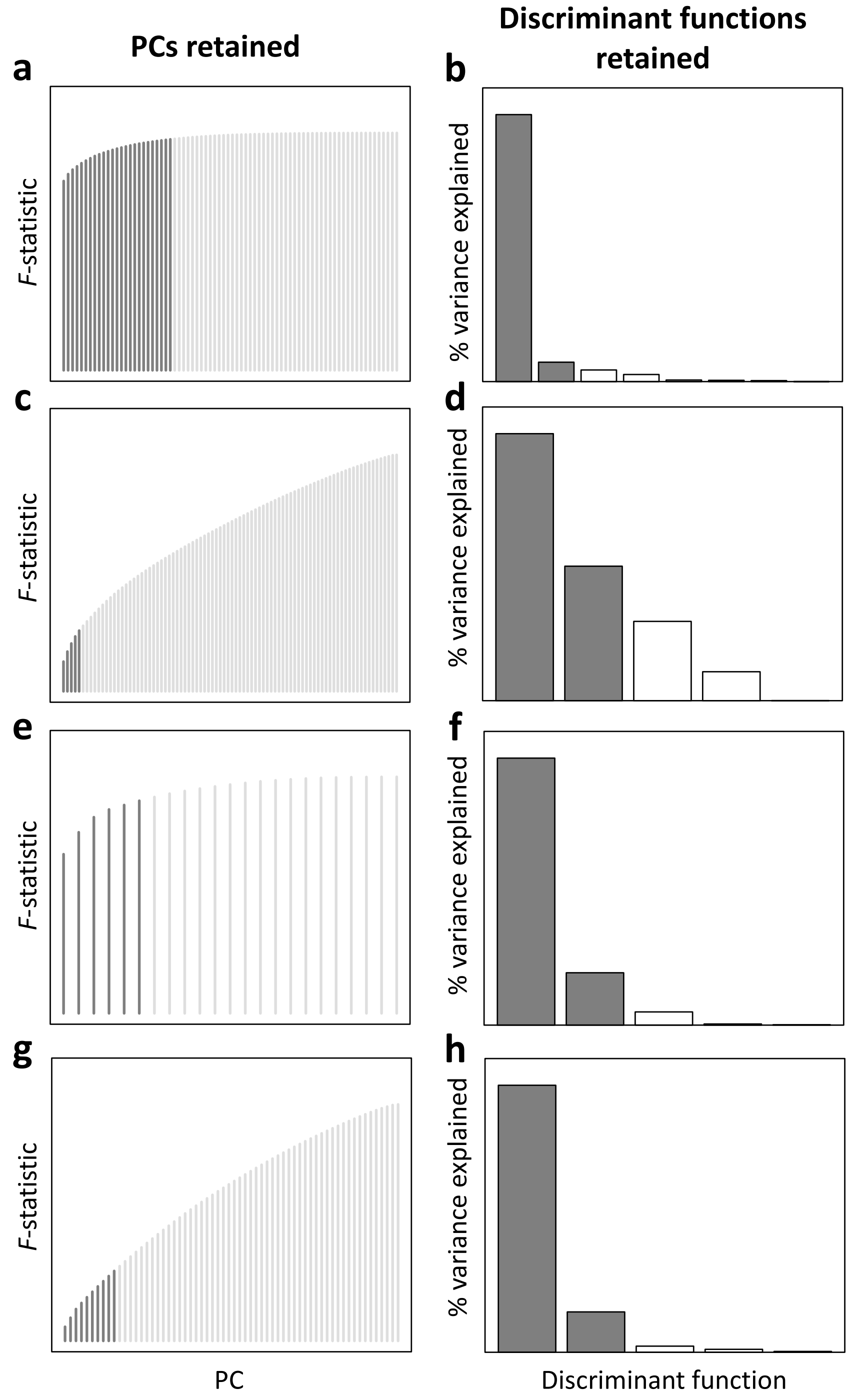

Suppl. Fig. 9. Number of principal components (PCs) (left column) and discriminant functions (right column) retained in discriminant analysis of principal component (DAPC) analysis for Smallmouth Bass (*Micropterus dolomieu*) and Neosho Bass (*M. velox*), with corresponding *F*-statistic and percent variance explained, respectively, for (a-b) outlier single nucleotide polymorphisms (SNPs) in all Smallmouth and Neosho Bass samples; (c-d) neutral SNPs in all Smallmouth and Neosho Bass samples; (e-f) outlier SNPs in Neosho Bass samples only; and (g-h) neutral SNPs in Neosho Bass only.

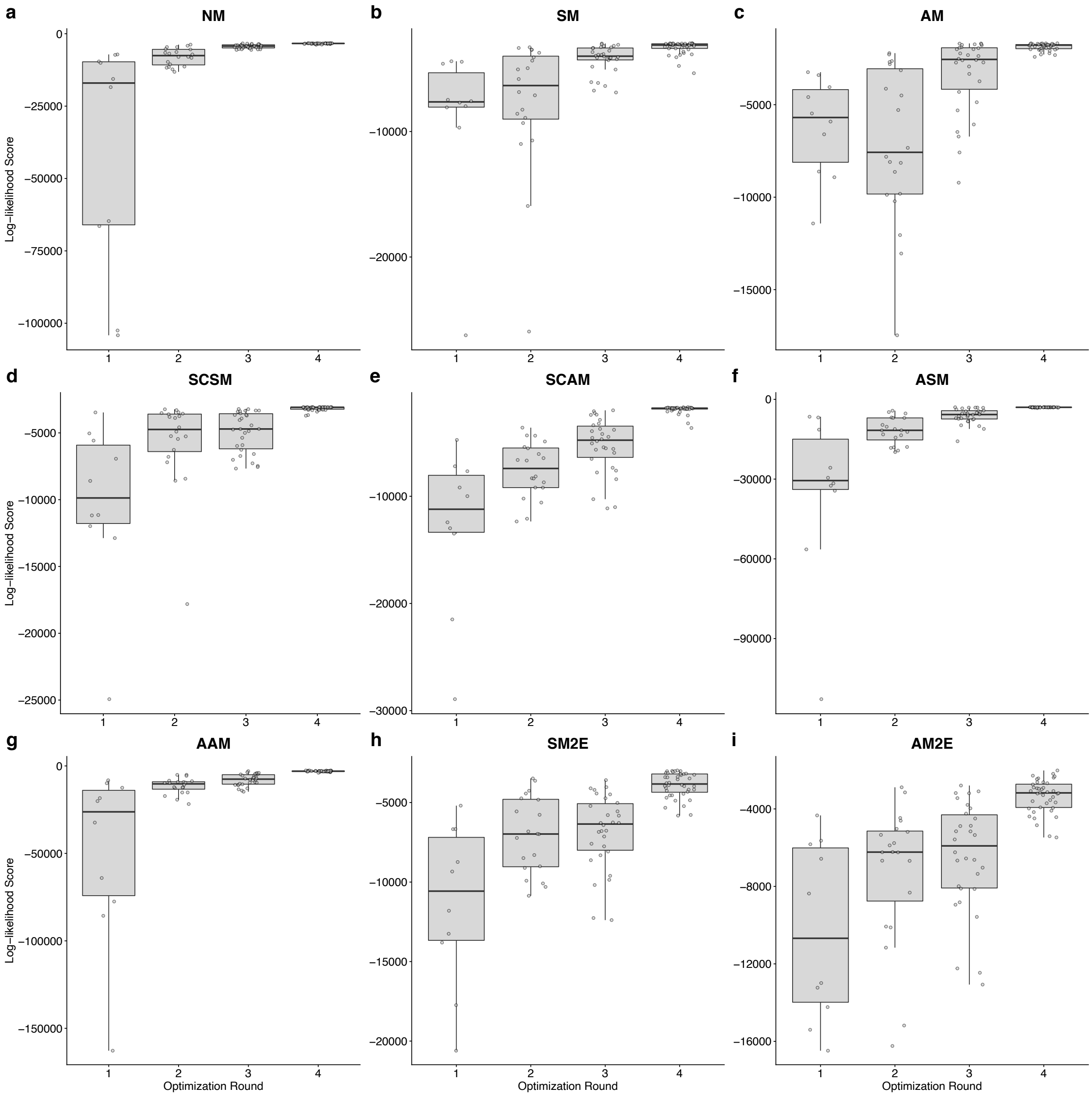

Suppl. Fig. 10. Optimization rounds for nine demographic models (a-i, corresponding to Table S2) run in *δaδi* for the admixed Neosho Bass (*Micropterus velox*) population in the Elk River (ELK) and its Smallmouth Bass parent (*M. dolomieu*) in the White River drainage (WHITE). Log-likelihood scores and associated box plots for 10 replicates in Round 1, 20 replicates in Round 2, 30 replicates in Round 3, and 40 replicates in Round 4, are shown for each model. Model codes are associated with the model descriptions in Table S2. Schematics for each model are given in Suppl. Fig. 2. Box plots depict the minimum, first quartile, median, third quartile, and maximum, with outliers depicted as single points.

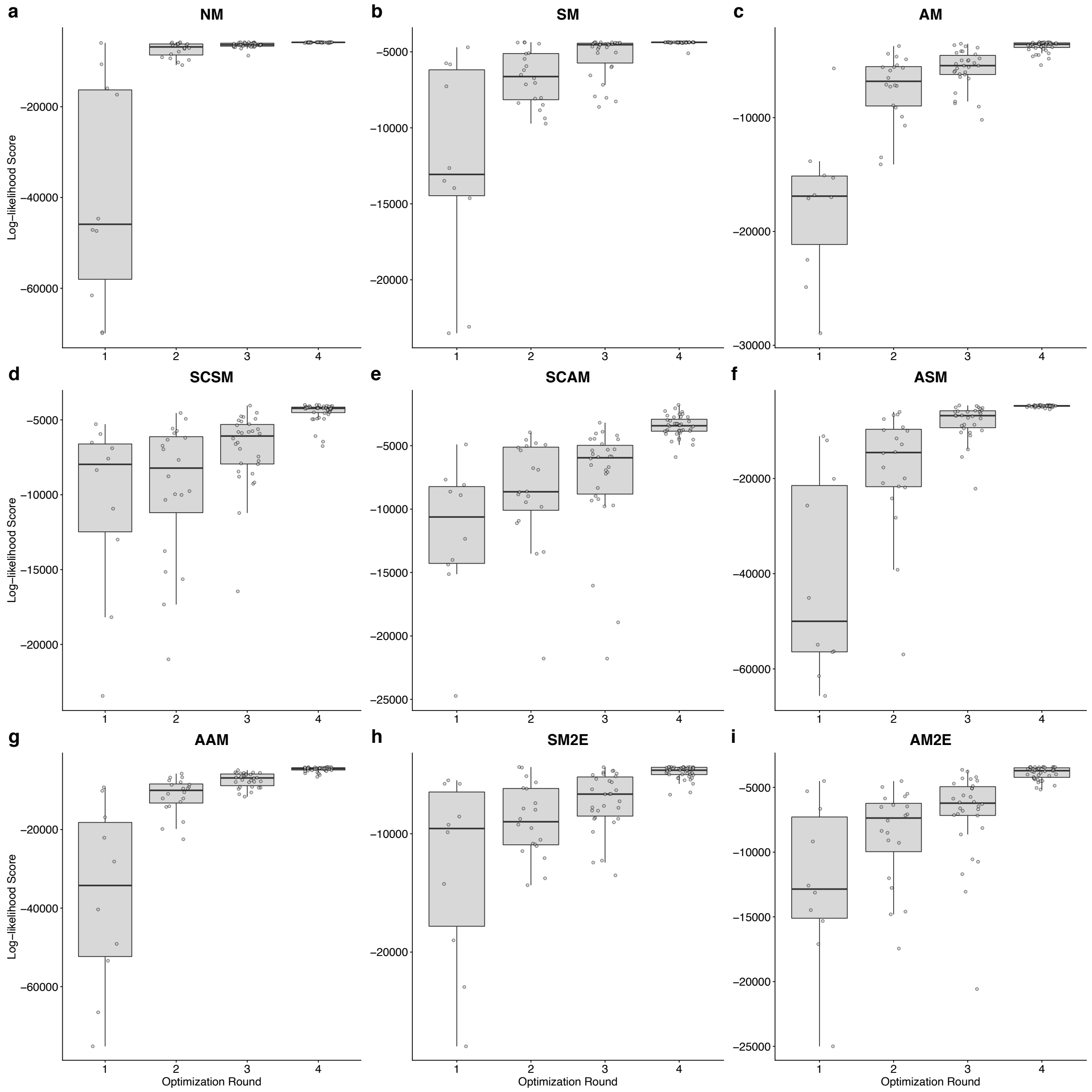

Suppl. Fig. 11. Optimization rounds for nine demographic models (a-i, corresponding to Table S2) run in *δaδi* for the admixed Neosho Bass (*Micropterus velox*) population in the Illinois River system (ILLI) and its Smallmouth Bass (*M. dolomieu*) parent in the hatchery strain represented by Skiatook Lake (SKIA). Log-likelihood scores and associated box plots for 10 replicates in Round 1, 20 replicates in Round 2, 30 replicates in Round 3, and 40 replicates in Round 4, are shown for each model. Model codes are associated with the model descriptions in Table S2. Schematics for each model are given in Suppl. Fig. 2. Box plots depict the minimum, first quartile, median, third quartile, and maximum, with outliers depicted as single points.

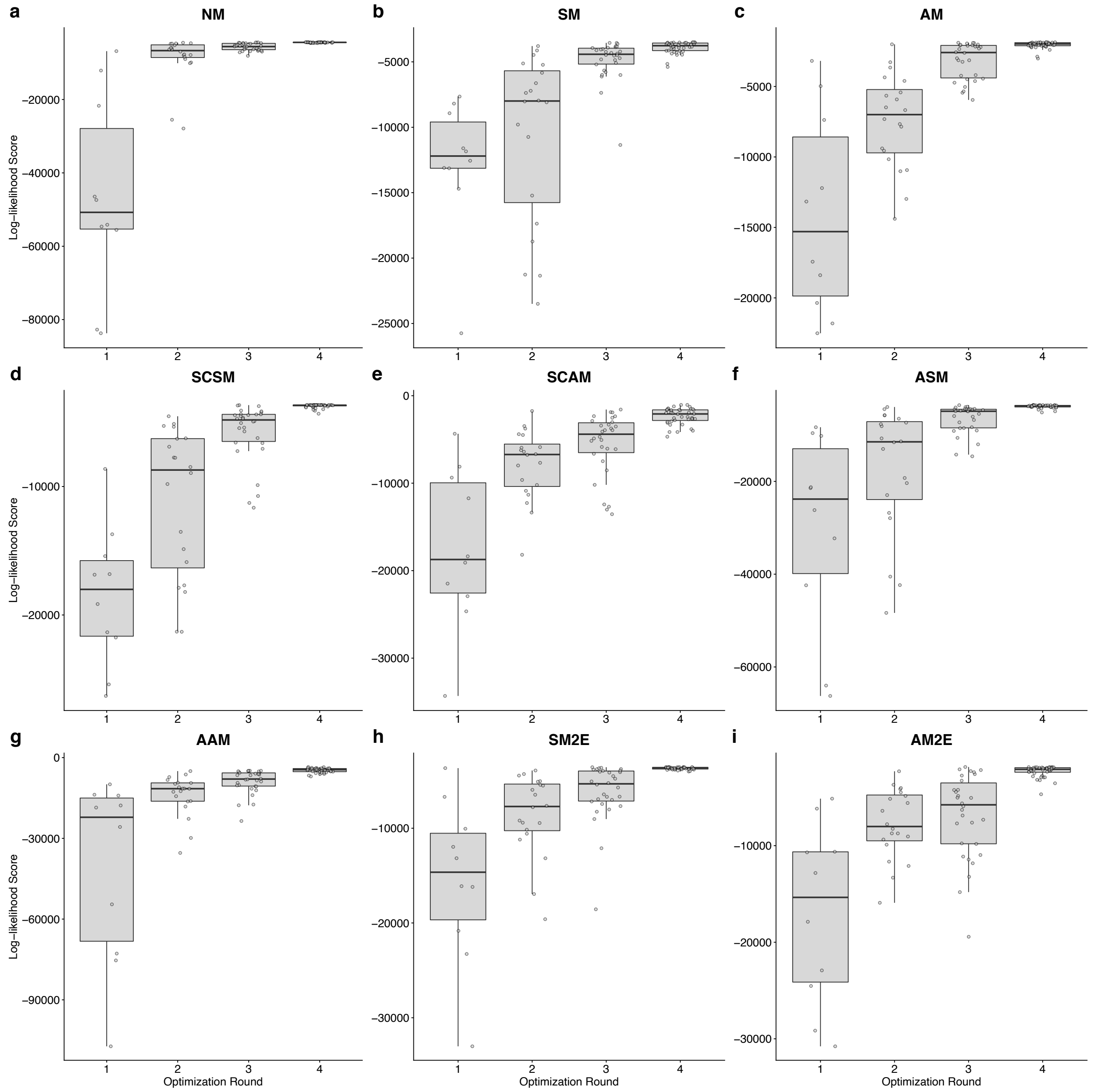

Suppl. Fig. 12. Optimization rounds for nine demographic models (a-I, corresponding to Table S2) run in *δaδi* for the admixed Neosho Bass (*Micropterus velox*) population in the Illinois Bayou River and Big Piney Creek, AR (BAYOU) and its Smallmouth Bass (*M. dolomieu*) parent in the White River drainage (WHITE). Log-likelihood scores and associated box plots for 10 replicates in Round 1, 20 replicates in Round 2, 30 replicates in Round 3, and 40 replicates in Round 4, are shown for each model. Model codes are associated with the model descriptions in Table S2. Schematics for each model are given in Suppl. Fig. 2. Box plots depict the minimum, first quartile, median, third quartile, and maximum, with outliers depicted as single points.

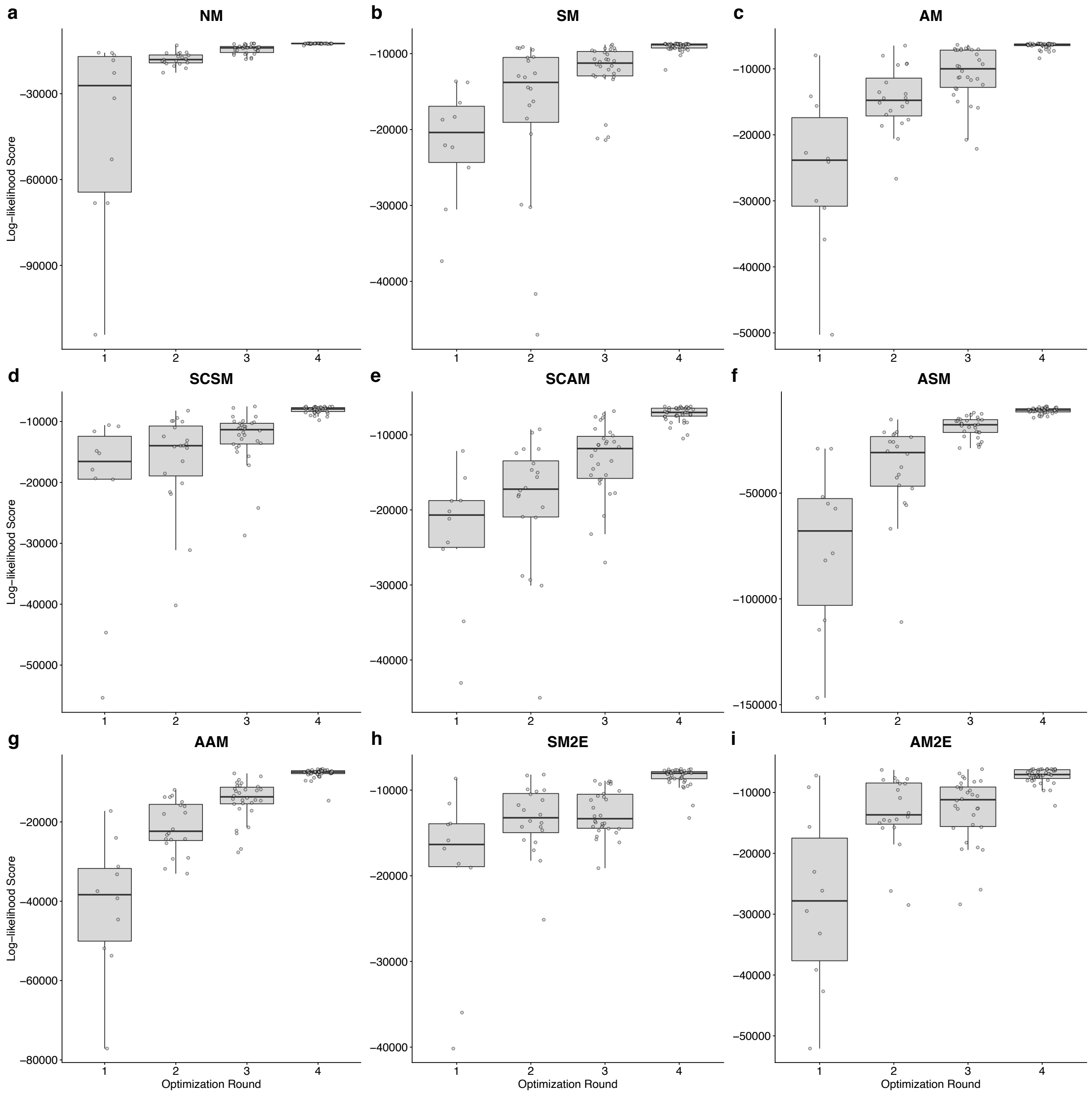

Suppl. Fig. 13. Optimization rounds for nine demographic models (a-i, corresponding to Table S2) run in *δaδi* for the admixed Neosho Bass (*Micropterus velox*) population in three streams of the upper Arkansas River Basin, including Buffalo Creek, Big Sugar Creek, and Spavinaw Creek (UPPARK) and its Smallmouth Bass parent (*M. dolomieu*) in the White River drainage (WHITE). Log-likelihood scores and associated box plots for 10 replicates in Round 1, 20 replicates in Round 2, 30 replicates in Round 3, and 40 replicates in Round 4, are shown for each model. Model codes are associated with the model descriptions in Table S2. Schematics for each model are given in Suppl. Fig. 2. Box plots depict the minimum, first quartile, median, third quartile, and maximum, with outliers depicted as single points.

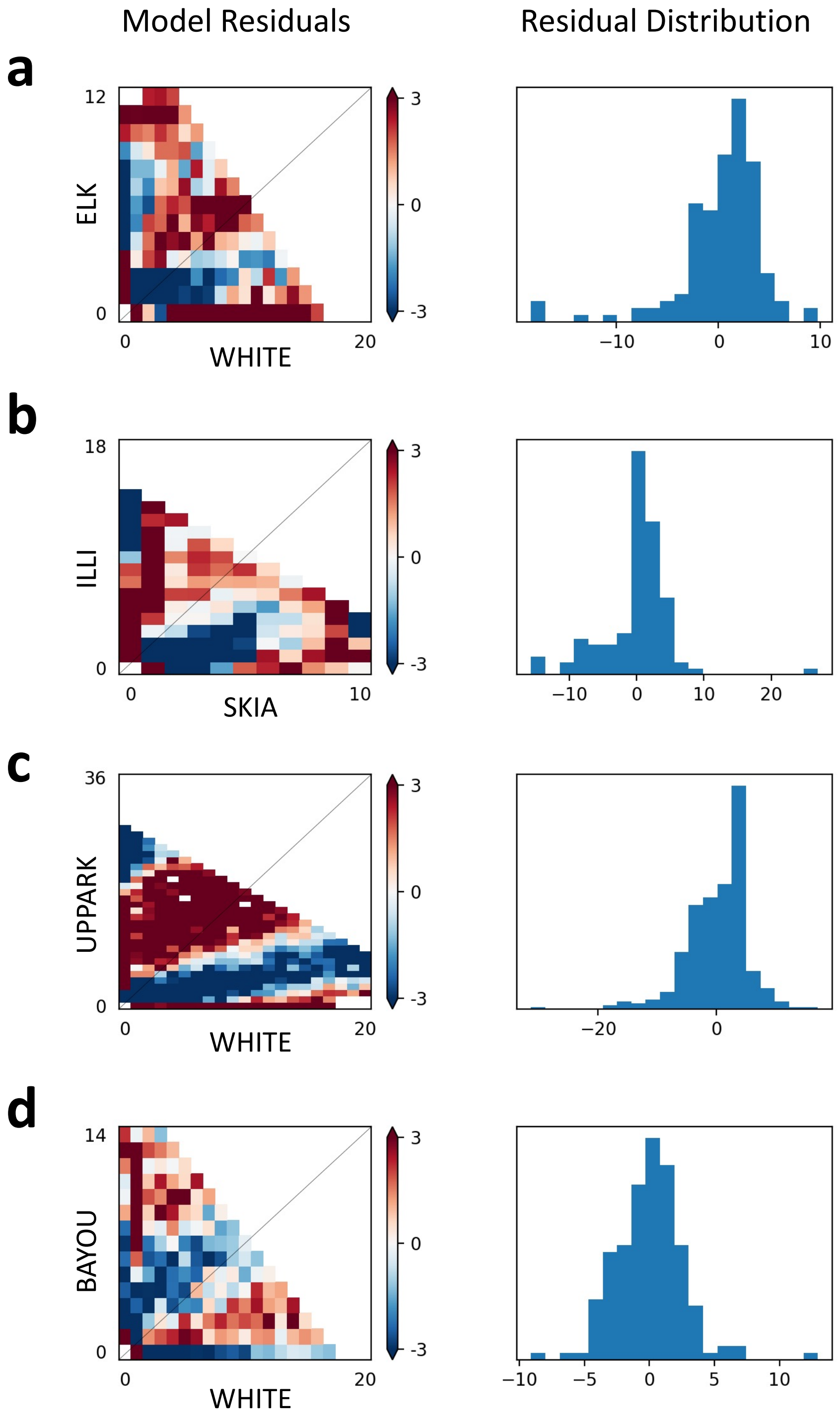

Suppl. Fig. 14. Residuals of empirical data (first column) and distribution of residuals (second column) with respect to the best fitting two-population demographic model for Smallmouth Bass (*Micropterus dolomieu*) and Neosho Bass (*M. velox*) populations, specifically for (a) ELK and WHITE, (b) ILLI and SKIA, (c) BAYOU and WHITE, and (d) UPPARK and WHITE.
